# Modeling food fortification contributions to micronutrient requirements in Malawi using Household Consumption and Expenditure Surveys

**DOI:** 10.1111/nyas.14697

**Published:** 2021-09-28

**Authors:** Kevin Tang, Katherine P. Adams, Elaine L. Ferguson, Monica Woldt, Alexander A. Kalimbira, Blessings Likoswe, Jennifer Yourkavitch, Benjamin Chrisinger, Sarah Pedersen, Lucia Segovia De La Revilla, Omar Dary, E. Louise Ander, Edward J. M. Joy

**Affiliations:** ^1^ Department of Population Health London School of Hygiene & Tropical Medicine London United Kingdom; ^2^ USAID Advancing Nutrition Arlington Virginia; ^3^ Institute for Global Nutrition University of California, Davis Davis California; ^4^ Helen Keller International Washington DC; ^5^ Department of Human Nutrition and Health, Faculty of Food and Human Sciences Lilongwe University of Agriculture and Natural Resources Lilongwe Malawi; ^6^ Department of Public Health, School of Public Health and Family Medicine University of Malawi Chichiri Malawi; ^7^ Results for Development Washington DC; ^8^ Department of Social Policy and Intervention University of Oxford Oxford United Kingdom; ^9^ USAID Bureau for Resilience and Food Security Washington DC; ^10^ USAID Bureau for Global Health Washington DC; ^11^ School of Biosciences University of Nottingham Loughborough United Kingdom; ^12^ Centre for Environmental Geochemistry British Geological Survey Keyworth United Kingdom

**Keywords:** large‐scale food fortification, HCES, micronutrient, inadequacy, equity, Malawi

## Abstract

Large‐scale food fortification may be a cost‐effective intervention to increase micronutrient supplies in the food system when implemented under appropriate conditions, yet it is unclear if current strategies can equitably benefit populations with the greatest micronutrient needs. This study developed a mathematical modeling framework for comparing fortification scenarios across different contexts. It was applied to model the potential contributions of three fortification vehicles (oil, sugar, and wheat flour) toward meeting dietary micronutrient requirements in Malawi through secondary data analyses of a Household Consumption and Expenditure Survey. We estimated fortification vehicle coverage, micronutrient density of the diet, and apparent intake of nonpregnant, nonlactating women for nine different micronutrients, under three food fortification scenarios and stratified by subpopulations across seasons. Oil and sugar had high coverage and apparent consumption that, when combined, were predicted to improve the vitamin A adequacy of the diet. Wheat flour contributed little to estimated dietary micronutrient supplies due to low apparent consumption. Potential contributions of all fortification vehicles were low in rural populations of the lowest socioeconomic position. While the model predicted large‐scale food fortification would contribute to reducing vitamin A inadequacies, other interventions are necessary to meet other micronutrient requirements, especially for the rural poor.

## Graphical abstract

Our analysis demonstrated the use of HCES data and a novel mathematical modeling framework to estimate the coverage, potential contributions, and equity dimensions of large‐scale food fortification in Malawi. While large‐scale food fortification was predicted to have high potential contributions in reducing vitamin A inadequacies throughout most of Malawi, additional interventions are likely required to meet the needs of rural populations of low SEP. This information provides relevant insight for nutrition policy and programs when designing strategies to alleviate the overall burdens of micronutrient deficiencies, although further research is necessary to identify the optimal combination of interventions for future micronutrient investments.

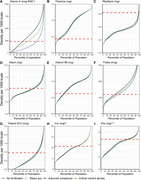

## Introduction

Micronutrient undernutrition burdens billions of people worldwide, disproportionately affecting the world's poorest countries and populations.^1^ Potential risks of micronutrient deficiencies can be characterized by estimating the prevalence of inadequate dietary intake, where individuals do not consume adequate quantities of bioavailable micronutrients to meet their physiological requirements.^2^ The global approach to estimating and understanding burdens of micronutrient undernutrition requires a combination of data types,^3^, ^4^ and dietary data play an essential role in identifying interventions that could provide sufficient additional micronutrients to populations with the greatest needs.^5^ The design of these interventions can be informed using mathematical models that combine food consumption data with food composition data to estimate total micronutrient contributions from the overall diet and the additional contribution of micronutrient interventions.^1^, ^6^, ^7^

One intervention that can increase dietary intake of micronutrients broadly across populations is large‐scale food fortification, where micronutrients are added to industrially processed, commonly consumed food items (also referred to as “fortification vehicles”) to increase each vehicle's micronutrient content.^8^ Large‐scale food fortification can be a cost‐effective intervention to increase the dietary supply of micronutrients in the food system.^9^ Mandatory fortification of food vehicles has been adopted into national policy by over 100 countries, with the national strategy depending on country context.^10^ Well‐implemented programs benefit from strong partnerships between the public and private sectors and guidance from the scientific community to maximize program contributions to improve micronutrient‐inadequate diets.

Household Consumption and Expenditure Surveys (HCESs) are a family of multicomponent socioeconomic surveys that collect detailed food consumption data, which, when combined with food composition data, can be used to estimate the household dietary supply of micronutrients.^11^ Before they can be used to assess the micronutrient adequacy of population diets, HCES food consumption data require transformation into usable metrics. One metric, the apparent intake approach, individualizes household micronutrient supplies by distributing micronutrients among household members in proportion to each individual's energy requirements.^12^ Micronutrient apparent intake can be standardized in reference to any individual, most commonly to adult males expressed as *adult male equivalents* (AME), but other household members can be set as the standardized reference, including nonpregnant, nonlactating, and premenopausal women expressed as *adult female equivalents* (AFE). Another metric, the micronutrient density approach, assesses the quality of the overall diet by estimating the total household micronutrient supply per unit of energy.^13^ Additional metrics, including fortification vehicle coverage and quantity of the vehicle consumed, can provide insights into the potential contribution of large‐scale food fortification toward meeting country's micronutrient needs.^14^

In Malawi, the diets of its near 19 million inhabitants depend on cereals, roots/tubers, and vegetables to supply a large proportion of micronutrient needs.^15^ Low consumption of micronutrient dense foods (e.g., animal‐sourced foods) and seasonal variation in the availability of certain fruits, vegetables, and root/tubers suggest that risks for dietary micronutrient inadequacy may be high and fluctuate throughout the year.^6^ The Malawian government has enacted the mandatory fortification of several fortification vehicles, which, when implemented together, are intended to fill micronutrient gaps throughout the population resulting from poor quality diets. This includes fortifying oil and sugar with vitamin A and wheat flour with vitamin A, thiamine, riboflavin, niacin, vitamin B_6_, folate, vitamin B_12_, iron, and zinc, with fortification occurring at large‐scale processing facilities.^16^ Over the past two decades, in parallel to the implementation of the number of micronutrient interventions,^17^ the prevalence of deficiencies of some micronutrients has substantially declined in Malawi.^18^ However, it is unknown how much can be attributable to large‐scale food fortification and the extent to which all populations are adequately served by current policies.

This study aimed to estimate the potential contributions of vitamin A–fortified oil and sugar and fortified wheat flour (with vitamin A, thiamine, riboflavin, niacin, vitamin B_6_, folate, vitamin B_12_, iron, and zinc) toward meeting dietary micronutrient requirements in Malawi using a mathematical modeling framework. The potential contribution of the large‐scale food fortification programs was measured according to the following objectives:

1. Coverage and apparent consumption quantity of each fortification vehicle;
2. The potential contribution of large‐scale food fortification to the micronutrient density of diets (or micronutrient supply per 1000 kcal of the population's diet), to apparent micronutrient intakes (per adult‐female equivalents), and to reduce the prevalence of dietary inadequacy among nonpregnant, nonlactating, premenopausal women;
3. Subpopulation differences in the adequacy of micronutrient density and apparent intake in urban and rural residences and between socioeconomic positions (SEPs).

Because Malawi mandates the fortification of three food vehicles with vitamin A, we estimated the potential contribution of each of the three food vehicles individually and in combination, while the potential contribution of the additional micronutrients in Malawi's wheat flour fortification standards was modeled individually. This study demonstrates the application of a mathematical modeling framework, which can be used in other country contexts, to estimate potential contributions of large‐scale food fortification, provided data are available. As the nutrition landscape in Malawi evolves, findings from this study can provide data‐driven inputs into policy discussions around Malawi's strategy to alleviate the burdens of micronutrient deficiencies.

## Materials and methods

### Model framework

The foundation for this model's framework requires data from HCES. While HCES questionnaires vary substantially between countries, this model's framework was designed to consider consistencies between HCES questionnaires from different countries so that the model can be applied in multiple contexts where HCES data are available. These similarities in HCES questionnaires include foods consumption recalled by the household over a fixed period, basic demographic data collected on each household member, and the inclusion of multiple questions to characterize an array of socioeconomic conditions.

The model in this study was based on HCES data from the Fourth Integrated Household Survey of Malawi (IHS4),^19^ conducted by the National Statistics Office of Malawi with support from the World Bank's Living Standards Measurement Study (LSMS). The IHS are used by the Government of Malawi to monitor the poverty and welfare of Malawian households. The IHS4 was implemented between April 2016 and April 2017, with a single‐visit 24‐module questionnaire collecting data on household living standards, expenditures, and other measures of social and economic welfare. The IHS4 sampling frame was based on the 2008 Malawi Population and Housing Census and was designed to be representative at national, urban/rural, regional, and district levels. Urban strata include the four major urban areas: Lilongwe City, Blantyre City, Mzuzu City, and the Municipality of Zomba, while rural strata include all 28 districts of Malawi (including the island district of Likoma). A stratified two‐stage sampling design was employed in which enumeration areas (*n* = 779) were selected at random to represent districts, with the probability of enumeration area selection proportional to size (i.e., the number of households), and 16 households were selected with equal probability from the total listing for each selected enumeration area. The final sample size was 12,447 households.

For this study, the IHS4 data were downloaded from the World Bank LSMS data portal (accessed September 28, 2020) and are used in compliance with the data access policy.^19^ The IHS4 data are deidentified at the source.

### Food consumption

The IHS4 included a food consumption module, in which a household member was asked to recall the quantity of foods consumed by the entire household in 7 days preceding the interview using a standardized list of 136 commonly consumed food items. The consumption quantities of each food item were reported in nonstandard units (e.g., sachets, pails, and pieces) with the help of visual aids. For this study, consumption quantities were converted to metric units, that is, kilograms. Food consumption quantities were adjusted for the nonedible portions of foods, for example, banana skins. The outlying values of consumption quantity for each food item (likely due to foods acquired in bulk or recall error^20^) were identified and replaced with the food item's median consumption quantity from the entire population. This provides a metric we refer to as *apparent consumption*.

### Micronutrient composition and large‐scale food fortification scenarios

This study modeled the contribution of the existing program of food fortification in Malawi, which mandates the fortification of cooking oil and sugar with vitamin A, and wheat flour with vitamin A, thiamine, riboflavin, niacin, vitamin B_6_, folate, vitamin B_12_, iron, and zinc. Food consumption data were matched with food composition data to estimate the household micronutrient supply of vitamin A, thiamine, riboflavin, niacin, vitamin B_6_, folate, vitamin B_12_, iron, and zinc. All food items were matched to an equivalent item from available food composition data. Data from the 2019 Malawian Food Composition Table (FCT)^21^ or from other food composition studies conducted in Malawi^22^ were prioritized for matching. For food items where data from Malawian sources were not available, FCTs from other eastern African countries^23^, ^24^, ^25^ were used as substitutes, and where data gaps remained, regional and international food composition databases^26^, ^27^, ^28^ were used. Food composition matches and references for all micronutrients and energy are available in Table S1 (online only).

Three scenarios were modeled and compared with an estimate of the potential contributions of oil, sugar, and wheat flour fortification programs to meet vitamin A needs and of wheat flour fortification to meet dietary requirements for the other eight micronutrients included in Malawi's wheat flour fortification standard (Table 1). First, the “no fortification” scenario was modeled, which assumed the food vehicles were not fortified with any micronutrients. This scenario provided estimates of the baseline (i.e., without fortification) adequacy of diets and served as a comparator to understand the potential contribution of Malawi's fortification program to improving the micronutrient adequacy of diets. Second, the “status quo” fortification scenario modeled large‐scale fortification at current levels of fortification, which are below the mandated standard levels. These levels, for each food fortification vehicle, were based on the analyzed micronutrient content of fortified food samples collected at sentinel sites in markets throughout Malawi in 2020.^29^ Third, the “improved compliance” scenario was modeled, which represented a hypothetical improvement in industry compliance to the national standards guidelines on average nutrient contents at the point of fortification, and adjusted for expected losses as summarized using the Food Fortification Formulator tool^30^ before preparation for consumption at home. This scenario resulted in higher micronutrient contents of each food vehicle compared with those modeled in the status quo scenario. For the status quo and improved compliance scenarios, the micronutrient composition of oil, sugar, and wheat flour was adjusted to create fortified products. The micronutrient composition of products made with wheat flour (e.g., bread, scones, and mandasi) was adjusted based on the proportional contents of wheat flour to reflect the three fortification scenarios (recipes and proportions are reported in Table S2, online only).

**Table 1:** Parameter values for food fortification modeling (composition per 100 g of a food item)

Micronutrient	Scenario	Cooking oil	Sugar	Wheat flour
Vitamin A (μg RAE)	No fortification	0	0	0
	Status quo*^a^*	1000	700	80
	Improved compliance*^b^*	2100	1050	180
Thiamine (mg)	No fortification	–	–	0.2
	Status quo	–	–	0.5
	Improved compliance	–	–	0.8
Riboflavin (mg)	No fortification	–	–	0.1
	Status quo	–	–	0.3
	Improved compliance	–	–	0.5
Niacin (mg)	No fortification	–	–	2.4
	Status quo	–	–	4.2
	Improved compliance	–	–	6.5
Vitamin B_6_ (mg)	No fortification	–	–	0.5
	Status quo	–	–	0.7
	Improved compliance	–	–	0.9
Folate (μg)	No fortification	–	–	240
	Status quo	–	–	427
	Improved compliance	–	–	648
Vitamin B_12_ (μg)	No fortification	–	–	0
	Status quo	–	–	0.6
	Improved compliance	–	–	1.3
Iron (mg)	No fortification	–	–	2.0
	Status quo	–	–	3.0
	Improved compliance	–	–	6.0
Zinc (mg)	No fortification	–	–	0.5
	Status quo	–	–	1.8
	Improved compliance	–	–	3.5

^*a*^ On the basis of food vehicle samples collected from sentinel sites in markets throughout Malawi.

^*b*^ On the basis of assuming industry compliance at point of fortification to meet national standards and accounting for fortificant deterioration during the time between production at the factory and preparation for consumption at the household.

### Data analysis

For objective 1, coverage was estimated as the percentage of households apparently consuming any quantity of the food vehicle. Apparent consumption was estimated as the quantity of each food vehicle consumed by households in grams per day, then divided by the number of AFE in each household,^12^ where expressing apparent consumption per AFE allows for comparison of apparent consumption across households of varying demographic composition. This method assumes that food consumed by the household is distributed within the household proportionally according to the energy requirements of each household member, standardized to a nonpregnant, nonlactating, 18‐ to 29‐year‐old female as the reference family member for reasons detailed in the following paragraph. The data and assumptions necessary to calculate the AFE factors are presented in Table S1 (online only). Coverage and apparent consumption estimates were calculated at the national level, then stratified by administrative regions and urban versus rural residences. Urban and rural subpopulations were further stratified into five SEPs on the basis of quintiles for total annual inflation–adjusted household expenditure per capita. These quintiles differed for urban and rural subpopulations.

For objective 2, the micronutrient density of the diet was estimated as a ratio of the household micronutrient supply to the total household energy supply and expressed per 1000 kcal for all three scenarios.^13^ A diet of inadequate density was defined as a household with a micronutrient density that fell below the critical nutrient density (CND) threshold,^13^ which is the ratio of an adult female's harmonized average requirement (H‐AR) (i.e., the average daily micronutrient intake that is estimated to meet the requirements of half of the healthy individuals and used to estimate inadequacy for populations)^31^ to an adult female's daily average energy requirement assuming moderate physical activity level.^32^ The nonpregnant, nonlactating 18‐ to 29‐year‐old woman was selected as the household reference as she has high micronutrient requirements relative to energy requirements compared with other household members and represents the average energy intake for the family. Therefore, if the household nutrient density was adequate to meet the micronutrient density requirements of an adult female, it is expected to meet the needs of most other household members of varying demographics. Households with micronutrient densities that fell below the CND threshold were classified as having an inadequate dietary micronutrient density. For the apparent intake approach for each micronutrient, dietary inadequacy was defined as the apparent intake per AFE below the micronutrient's H‐AR for an adult female.^31^ Since nonpregnant, nonlactating 18‐ to 29‐year‐old women were selected as the household reference to define the CND threshold due to higher density requirements compared with other household members, apparent intakes were expressed per AFE (rather than per AME) in order to maintain the consistency between micronutrient density and apparent intake inadequacy thresholds. Table S3 (online only) shows the CNDs and H‐AR thresholds for adult females for each micronutrient used to define these two dimensions of dietary inadequacy.

For objective 3, differences in dietary inadequacy assessed using the micronutrient density and apparent intake approaches were analyzed between SEPs in urban and rural residences under all three fortification scenarios. To visualize seasonal patterns in the household micronutrient supply over time, the micronutrient density and apparent intake estimates were assessed by survey date for each subpopulation for all micronutrients using a weighted least‐squares local regression (“loess”) to reduce the effect of outliers.^33^ Loess smoothing used quadratic equations to fit within each “moving window,” where α = 0.75. Seasonality curves and 95% confidence intervals were presented for each of the fortification scenarios and compared in relation to the CND for the micronutrient density seasonality curves and the H‐AR for the apparent intake seasonality curves.

Data analysis was conducted in RStudio® (version 3.6.1, the R Foundation for Statistical Computing), using a variety of elements in the *tidyverse* package,^34^ including *dplyr* for data cleaning and transformation and *ggplot2* for visualization. Detailed descriptions of the data cleaning and transformation procedures are available in the file Supplementary Materials (online only), and, in addition, further analysis methods and code are available upon request.

## Results

### Description of the population

All 12,447 households surveyed in the IHS4 were included in the analysis, where 10,175 (82%) were rural, and 2272 (18%) were urban. Characteristics of the urban, rural, and SEP subpopulations are presented in Table 2. Compared with urban households, rural households were more likely to be farmers, less likely to be in wage employment, had lower completion rates of formal education for both men and women, were more likely to participate in cash transfer and nutrition social safety net programs, and were more likely to have at least one wasted, stunted, or underweight child in the household. Differences in these characteristics were even more pronounced between rural households of the lowest and highest SEPs.

**Table 2:** Descriptive summary of the survey population from the Fourth Integrated Household Survey of Malawi (2016/17)

Residence	Rural					Urban					
The socioeconomic position by quintile of total annual household expenditure	Lowest	Lower‐middle	Middle	Upper‐middle	Highest	Lowest	Lower‐middle	Middle	Upper‐middle	Highest	*P* value (urban/rural)*^a^*
Households, *n*	2035	2035	2035	2035	2035	455	454	455	454	454	–
Anyone in household's main occupation is…											
Wage employment, %	3	6	8	10	21	29	42	51	57	72	<0.001
Household business (nonagriculture), %	8	10	12	14	20	28	43	47	43	33	<0.001
Household agriculture/farming, %	89	90	87	84	72	49	36	27	24	13	<0.001
Distance (km) to the nearest:											
Road, mean	12.5	11.5	10.9	10.9	9.7	2.8	2.3	2.8	2.4	1.6	<0.001
Agricultural market, mean	24.1	24.7	26.2	27.2	26.9	8.4	7.4	7.6	7.3	5.4	<0.001
Population center, mean	41.8	41.2	40.7	41.0	41.1	14.7	11.6	11.3	10.6	9.5	<0.001
Highest educational qualification (males)											<0.001
None, %	69	63	54	51	39	52	36	24	17	8	
Primary school, %	15	18	23	23	24	31	33	30	20	11	
Secondary school +, %	2	3	6	8	18	12	24	36	53	64	
Highest educational qualification (females)											<0.001
None, %	80	72	67	60	44	64	44	31	17	8	
Primary school, %	10	15	18	22	23	25	41	40	34	14	
Secondary school +, %	1	2	2	3	10	4	8	20	37	55	
Participate in social safety net programs											
Cash transfers, %	6	6	5	5	4	3	2	1	0	0	<0.001
Nutrition programs, %	41	39	37	35	27	27	26	21	13	3	<0.001
At least one child under 5 in household, *n*	1270	1038	904	824	602	262	229	202	145	83	
Wasted (WHZ<−2) or edematous (apparent), %*^b^*	11	8	9	6	8	10	6	7	5	5	<0.001
Stunted (HAZ<−2), %*^b^*	32	31	32	30	27	29	27	25	23	17	<0.001
Underweight (WAZ<−2), %*^b^*	13	11	12	8	9	8	6	8	5	5	<0.001

^*a*^ Urban/rural differences within variables tested using Pearson's Chi‐squared test for factor variables and Student's *t*‐test for numeric variables.

^*b*^ Among households *with* at least one child under 5 years.

HAZ, height‐for‐age Z‐score; WAZ, weight‐for‐age Z‐score; WHZ, weight‐for‐height Z‐score.

### Objective 1: Fortification vehicle coverage and apparent consumption

Table 3 presents the coverage of each fortification vehicle, or the percentage of households apparently consuming any quantity of the fortification vehicles during the 7‐day recall period. Nationally, 76% of households reported consuming oil, and 56% reported consuming sugar. The percentage of households consuming any quantity of wheat flour or wheat flour products was 52% and varied depending on the product: 35% consumed mandasi/doughnut, 22% consumed bread, and 14% consumed buns/scones. Coverage of all fortification vehicles was lower in rural populations compared with urban populations, and coverage of oil and sugar in urban areas was nearly universal (96% and 92% of households, respectively). Coverage decreased with SEP, with the lowest coverage observed in the poorest populations in both urban and rural residences for the three fortification vehicles. In rural populations, when comparing the lowest to highest SEPs, coverage of sugar was 17% of households in the lowest quintile compared with 80% in the highest quintile, and coverage of wheat flour was 20% in the lowest quintile compared with 73% in the highest quintile. Coverage between Malawi's three administrative regions was similar, ranging between 74% and 79% for oil, 52% and 66% for sugar, and 50% and 55% for wheat flour.

**Table 3:** Percentage of households apparently consuming any or none of the food fortification vehicles and median consumption quantity among consumers (grams per adult female equivalents per day) from the Fourth Integrated Household Survey of Malawi

	Households	Oil		Sugar		Wheat flour and products		None consumed
Population	*n*	*%*	Median (IQR)	%	Median (IQR)	%	Median (IQR)	%
National (total)	12,447	76	12 (5, 23)	56	28 (19, 40)	52	9 (4, 28)	17
Geography by administrative region								
North	2491	79	14 (9, 24)	66	31 (22, 45)	50	13 (5, 33)	14
Center	4220	74	9 (3, 20)	55	28 (20, 40)	55	7 (3, 28)	17
South	5736	76	13 (5, 24)	52	26 (17, 38)	50	10 (4, 27)	18
Residence and socioeconomic position (SEP) by quintile of total annual household expenditure per capita								
Rural (total)	10,175	72	10 (4, 19)	48	25 (17, 37)	44	6 (3, 16)	20
Lowest SEP	2035	44	3 (1, 8)	17	11 (6, 19)	20	2 (2, 4)	45
Lower middle SEP	2035	66	6 (2, 12)	34	18 (10, 24)	33	3 (2, 6)	25
Middle SEP	2035	75	9 (3, 15)	47	21 (14, 29)	42	4 (3, 8)	16
Upper middle SEP	2035	83	11 (5, 20)	62	26 (19, 37)	53	6 (3, 15)	10
Highest SEP	2035	91	19 (11, 33)	80	36 (26, 51)	73	16 (6, 34)	3
Urban (total)	2272	96	21 (12, 34)	92	34 (25, 48)	86	29 (14, 53)	2
Lowest SEP	455	87	9 (4, 14)	76	23 (16, 30)	64	7 (3, 18)	6
Lower middle SEP	454	97	15 (9, 23)	92	28 (22, 39)	84	19 (9, 33)	1
Middle SEP	455	98	22 (15, 31)	97	36 (27, 48)	92	29 (15, 43)	0
Upper middle SEP	454	98	26 (18, 37)	98	40 (30, 51)	95	40 (23, 62)	0
Highest SEP	454	99	39 (27, 62)	97	45 (31, 67)	94	58 (34, 87)	0

IQR, interquartile range.

Table 3 also presents the median apparent consumption of each food vehicle. Nationally, the median apparent consumption was 12 g/day per AFE for oil, 28 g/day per AFE for sugar, and 9 g/day per AFE for wheat flour. For all three vehicles, the median apparent consumption was higher among urban households than rural households. For all three food vehicles in both rural and urban residences, apparent consumption increased as the SEP increased. In rural populations: from 3 to 19 g/day per AFE for oil, from 11 to 36 g/day per AFE for sugar, and 2–16 g/day per AFE for wheat flour. In urban populations: from 9 to 39 g/day per AFE for oil, from 23 to 45 g/day per AFE for sugar, and 7–58 g/day per AFE for wheat flour. Median apparent consumption between administrative regions ranged from 9 to 14 g/day per AFE for oil, 26–31 g/day per AFE for sugar, and 7–13 g/day per AFE for wheat flour.

### Objective 2: Micronutrient inadequacy

**Figure 1:**
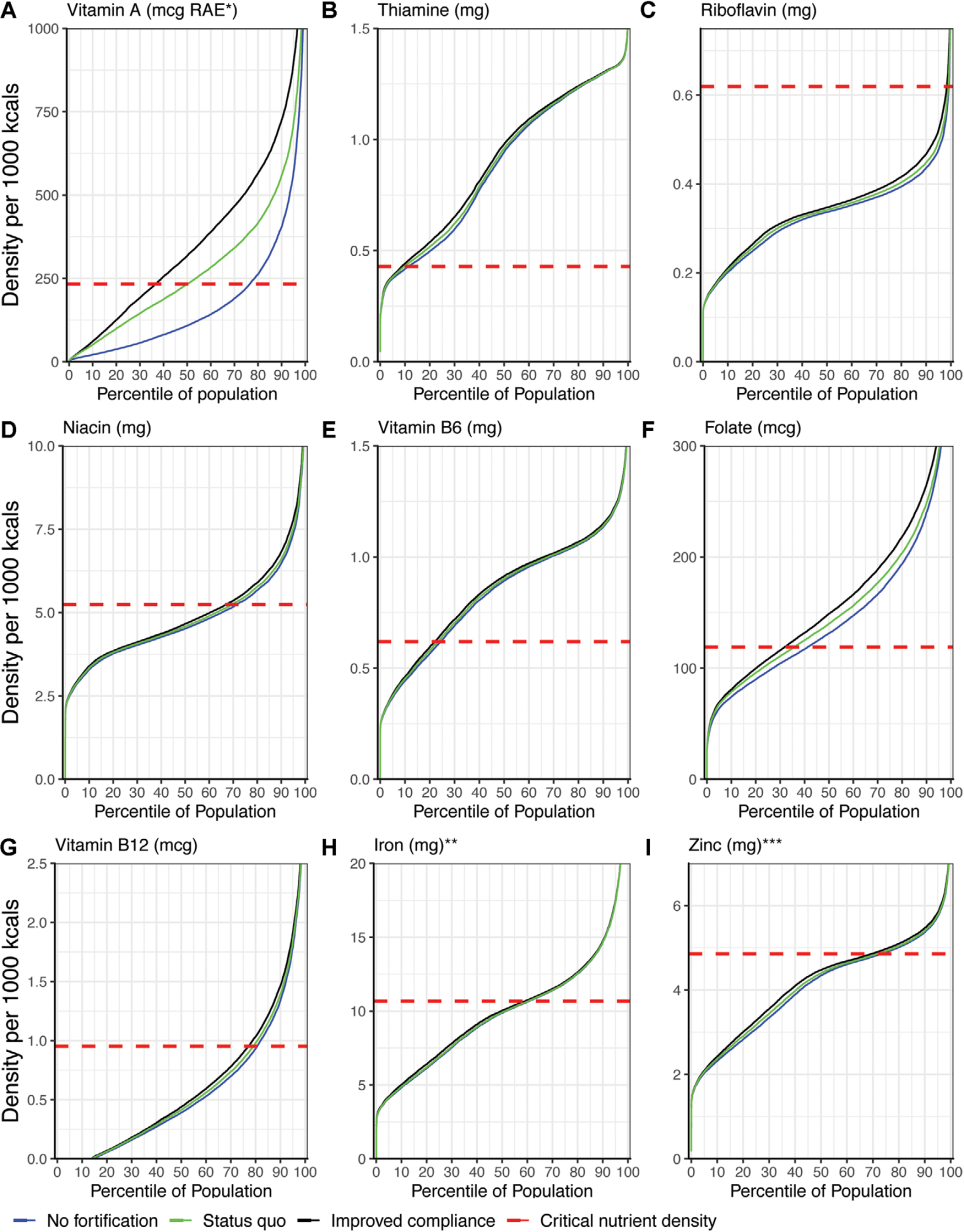
Micronutrient density population percentile curves and estimates of dietary inadequacy for three fortification scenarios for (A) vitamin A, (B) thiamine, (C) riboflavin, (D) niacin, (E) vitamin B_6_, (F) folate, (G) vitamin B_12_, (H) iron, and (I) zinc. ^*^RAE, retinol activity equivalents. ^*^^*^Iron inadequacy cannot be defined using the critical nutrient density cut‐point method due to the nonnormal distribution of iron requirements. ^*^^*^^*^Zinc inadequacy thresholds assume low bioavailability because of a diet high in unrefined grains.

Micronutrient density percentile curves modeling the three fortification scenarios are shown in Figure 1. Under the no fortification scenario, micronutrients with a high prevalence of inadequate density (more than 75%) were vitamin A, riboflavin, and vitamin B_12_; the moderate prevalence of inadequate density (between 50% and 75%) were niacin, iron, and zinc; and low prevalence of inadequate density (between 25% and 50%) was folate. The inadequate density of thiamine and vitamin B_6_ was very low (<25%).

**Figure 2:**
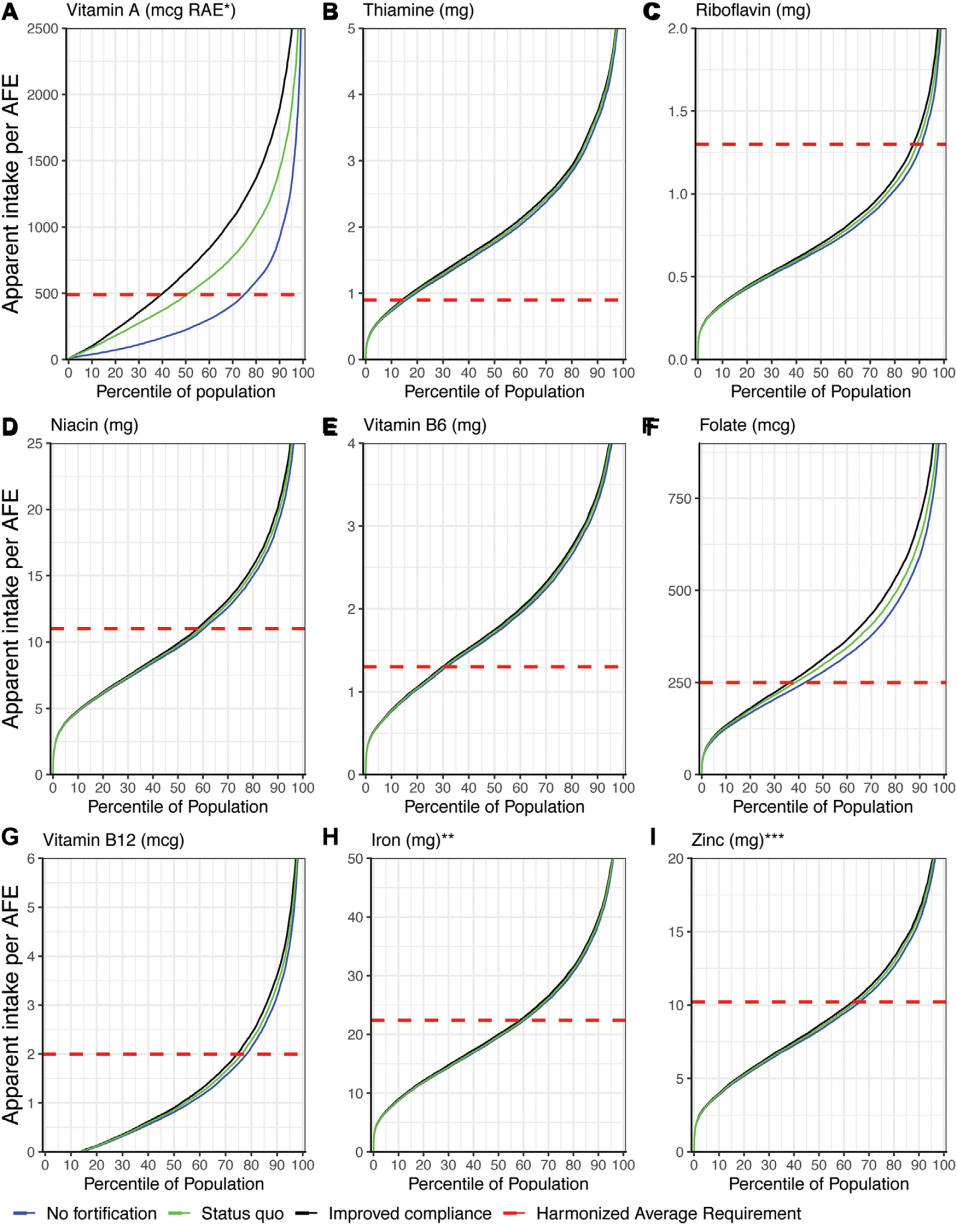
Micronutrient apparent intake population percentile curves and estimates of dietary inadequacy for three fortification scenarios for (A) vitamin A, (B) thiamine, (C) riboflavin, (D) niacin, (E) vitamin B_6_, (F) folate, (G) vitamin B_12_, (H) iron, and (I) zinc. ^*^RAE, retinol activity equivalents. ^*^^*^Iron inadequacy cannot be defined using the critical nutrient density cut‐point method due to the nonnormal distribution of iron requirements. ^*^^*^^*^Zinc inadequacy thresholds assume low bioavailability due to a diet high in unrefined grains.

Under the status quo fortification scenario, only the prevalence of the inadequate density of vitamin A and folate was reduced. A reduction in the inadequate density of vitamin A (Fig. 1A) was mainly due to the combined contributions from oil and sugar, where wheat flour also provided additional vitamin A but in negligible amounts. There was potential for further declines in the prevalence of inadequate density for vitamin A and folate if the average content of each food vehicle met the current Malawian regulations via improved compliance. Improved compliance of wheat flour fortification did not reduce the prevalence of inadequate density for all the other micronutrients due to the low consumption of wheat flour in Malawi. The results for micronutrient inadequacy estimated by apparent intake per AFE were similar to inadequate density estimates using the micronutrient density approach compared with the CND threshold, and the equivalent figures are shown in Figure 2.

### Objective 3: Comparison between urban, rural, and socioeconomic strata

**Figure 3:**
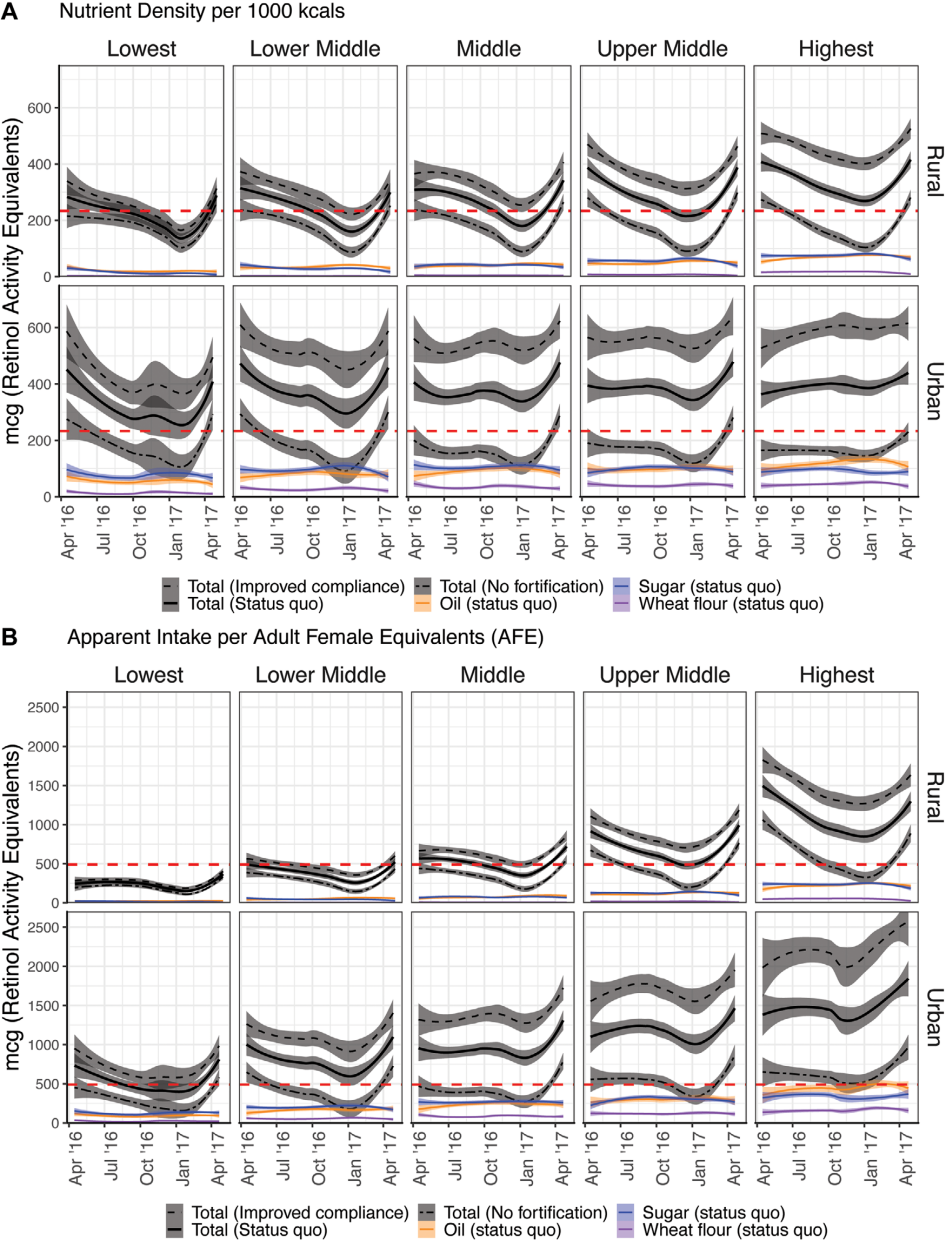
Seasonality in the (A) nutrient density and (B) apparent intake of vitamin A under the three fortification scenarios in relation to the inadequacy threshold (red dotted line) by socioeconomic position (lowest to highest) between urban and rural residences. Panels of seven further micronutrients are available in Supplementary Figures S1–S7 (online only).

Figure 3 presents the vitamin A supply graphs comparing the no fortification, status quo, and improved compliance scenarios by season and disaggregated by urban, rural, and socioeconomic subpopulations. Deductions on seasonal contributions of vitamin A fortification between subpopulations were similar when comparing the micronutrient density approach (Fig. 3A) versus the apparent intake approach (Fig. 3B). Vitamin A–fortified oil and sugar had the potential to decrease vitamin A inadequacy in most strata, except rural populations of low SEP. However, populations of high SEP benefit the most from fortification because, in these populations, fortification increased the vitamin A density of the diet at greater margins compared with populations of low SEP (Fig. 3A), and fortification vehicles were consumed in higher quantities (Fig. 3B). Under the improved compliance scenario, rural populations of low SEP still had inadequate vitamin A density during seasons where vitamin A supply was the lowest despite higher fortification contents.

Apparent intake of vitamin A (Fig. 3B) under the no fortification scenario indicates that populations of low SEP in both rural and urban residences would be expected to have an inadequate apparent intake of vitamin A across seasons, mostly due to the lower apparent intakes of the fortification vehicles compared with populations of higher SEP rather than lower vitamin A density of their diets. Vitamin A contributions from oil, sugar, and, to a lesser degree, wheat flour remained consistent throughout the year, demonstrating little seasonal fluctuation. Consistent vitamin A supply across seasons from fortification vehicles contrasted the large seasonal fluctuation of vitamin A supply from the nonfortified diet, suggesting that fortified foods may be able to help fill gaps in seasonal inadequacy from natural food sources in the diet. In summary, total vitamin A apparent intake increased in all urban and rural populations of high SEP when compared with the no fortification scenario. Rural populations of low SEP saw minimal contributions from fortification under both the status quo and improved compliance scenarios.

**Figure 4:**
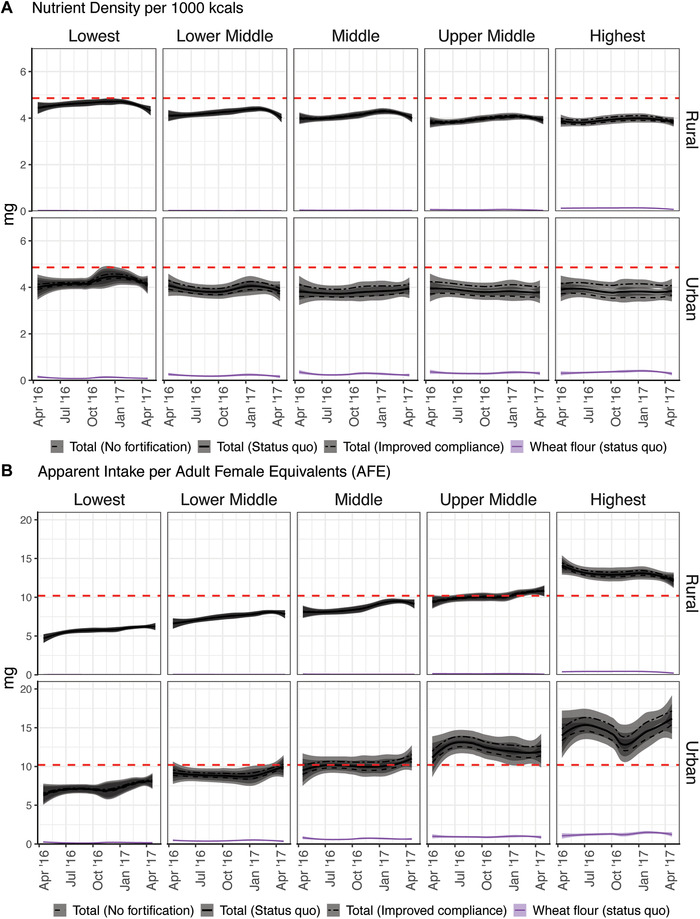
Seasonality in the (A) nutrient density and (B) apparent intake of zinc under the three fortification scenarios in relation to the inadequacy threshold (red dotted line) by socioeconomic position (lowest to highest) between urban and rural residences. Panels of seven further micronutrients are available in Supplementary Figures S1–S7 (online only).

Figure 4 shows seasonality in zinc micronutrient density (Fig. 4A) and apparent intake (Fig. 4B) in urban and rural populations across SEPs. Despite their low zinc concentration, cereals contributed the largest proportion of zinc to the total dietary supply, and micronutrient density of the diet decreased as the SEP increased. However, zinc apparent intake increased as the SEP increased, indicating that populations of higher SEP are consuming more food overall. Wheat flour was the sole fortification vehicle for zinc, and unlike the case with vitamin A, fortification did not lead to substantial changes in total dietary zinc supply, and inadequacy in the two fortification scenarios remained similar to the no fortification scenario in all groups with the exception of those of the highest SEP.

Figures S1 through S7 (online only) present seasonality plots disaggregated by urban, rural, and socioeconomic subpopulations for all other micronutrients provided via wheat flour fortification. For all micronutrients measured using both the micronutrient density and apparent intake, the no fortification, status quo, and improved compliance scenarios were similar in all subpopulations, with the exception of the urban populations of high SEP, which received marginal contributions from wheat flour fortification.

## Discussion

In this paper, we present an application of a mathematical modeling framework using open‐source HCES data to model the coverage, potential contributions, and equity dimensions of the fortification of oil, sugar, and wheat flour for a range of micronutrients in Malawi. Our analysis showed that oil and sugar had wide coverage (>50% of households), and fortification was predicted to reduce the prevalence of vitamin A inadequacy of diets (75% inadequate density under the no fortification scenario versus 50% under the status quo scenario, and 37% under the improved compliance scenario). Wheat flour also had wide coverage (52% at the national level, 44% in rural areas, and 86% in urban areas), but low apparent consumption across the population resulted in low potential contributions for all micronutrients, with little potential for improvement through improved industry compliance alone. All fortification vehicles demonstrated lower coverage and potential contributions in rural populations of the lowest SEP, suggesting that additional micronutrient interventions are necessary to meet the micronutrient needs of these most vulnerable groups.

### Oil and sugar fortification

In Malawi, wide coverage and adequate consumption quantities make oil and sugar good candidates for vitamin A fortification vehicles. Vegetables and fruits contribute the largest proportion of vitamin A to the unfortified Malawian diet,^15^ but the availability of these foods fluctuates seasonally (Fig. 3). This analysis found very little variation in oil and sugar consumption across seasons, suggesting that fortification of these products can provide a consistent source of vitamin A during times of the year when dietary supply is otherwise low. Vitamin A contributions from fortified oil and sugar can increase vitamin A density of diets to adequate levels in most urban and some rural populations, especially if compliance to industry standards at point of fortification is met. Strategies to improve compliance could include investment in improved government regulatory monitoring,^35^ subsidies to producers for internal product quality control and reporting, and improved fortified product labeling to improve the feasibility of spot‐checks throughout the food value chain.^36^

Rural populations of the lowest SEP do not consume enough oil and sugar to sufficiently benefit from industrial fortification, suggesting that the current fortification interventions alone are not sufficient to meet the vitamin A requirements for the entire population of Malawi. These findings suggest that to alleviate vitamin A inadequacies successfully and sustainably, additional micronutrient interventions are required that target these hard‐to‐reach populations who have the highest prevalence of vitamin A inadequacy. There are many other interventions with demonstrated success in improving vitamin A intake across populations. First, vitamin A–rich crop varieties through biofortification (i.e., orange‐fleshed sweet potato, orange maize, and orange cassava prototypes) in combination with a comprehensive behavior change communication strategy to promote dietary diversification could increase vitamin A supply to these groups if programs are designed to reach vulnerable populations.^37^ Second, for preschool children (PSC), Malawi's high‐dose vitamin A supplementation program was reported to have high coverage (67% received a supplement in the past 6 months) with an equal reach between rural and urban populations and across all SEPs.^38^ Recent evidence suggests that the combination of vitamin A fortification and supplementation interventions may be leading to excessive vitamin A intake among PSC in Malawi,^18^ although caution is needed in interpreting these data and more research is needed before consideration of any policy changes.^39^, ^40^ We found a low risk of excessive vitamin A intakes under the status quo fortification scenario, although the risk of excessive apparent intake increases to 19% among wealthy, urban households under the improved compliance scenario (Table S5, online only). Further research is needed to model how additional micronutrient interventions can meet vitamin A needs unaddressed by oil and sugar fortification.

### Wheat flour fortification

The fortification of wheat flour with nine micronutrients in Malawi is predicted to have minimal effect on reducing the prevalence of dietary micronutrient inadequacy. When consumed, wheat flour products are not consumed in high quantities in Malawi outside of urban populations of high SEP and increasing wheat flour fortification to meet regulatory standards was shown to have little effect on the estimated prevalence of inadequacy. Since this analysis indicates that micronutrient requirements are not being met through the diet alone (especially for riboflavin, niacin, vitamin B_12_, iron, and zinc), additional micronutrient interventions are likely necessary to meet dietary requirements.

For example, agronomic biofortification (i.e., enriching mineral concentration of crops through the application of mineral fertilizers) could be effective in increasing the supply of minerals from staple crops and improving yields on infertile soils.^41^, ^42^ Other varieties of iron‐ and zinc‐enriched biofortified cereals and legumes (e.g., beans and pearl millet) have the potential to increase micronutrient supplies from food items commonly consumed in Malawi.^43^ For industrial fortification, maize flour has wider coverage and higher quantity consumption throughout Malawi,^6^, ^38^ but decentralized milling via thousands of small and medium enterprises posed significant mechanical, financial, and quality control challenges when a large community‐level fortification program was implemented in Malawi from 1998 to 2009.^44^ Further research is needed to assess the potential contribution of these alternative interventions to meeting micronutrient needs and the cost‐effectiveness of Malawi's wheat flour fortification program relative to, and in combination with, other micronutrient programs.

### Relevance for national nutrition strategies

While identifying micronutrient interventions that can reach vulnerable populations with the greatest micronutrient gaps is important, efforts to improve the micronutrient quality of the diet (estimated using the nutrient density metric) should be balanced with efforts to ensure that these populations have adequate quantities of micronutrients (estimated using the apparent intake metric), that is to ensure that households have both adequate supplies of energy and supplies of micronutrient dense foods. Similar micronutrient densities of the diet between subpopulations (assuming no fortification), yet lower apparent intake in the rural populations of the lowest SEP, suggest that the quality of the family diet does not vary greatly throughout the whole country, but rural populations of the lowest SEP have inadequate energy intakes. The apparent energy intake of the poorest 40% of rural populations was below 1636 kcal/day,^15^ which is noticeably below the 2100 kcal/day recommended mean daily energy intake for an adult female with moderate physical activity levels.^32^ While large‐scale food fortification can fill some micronutrient gaps, a continued investment in improving the food security of these vulnerable populations is necessary.

The availability of multiple types of nutrition data requires careful integration when assessing the micronutrient status of a population. A variety of data types (e.g., micronutrient biomarker, household consumption and expenditure, individual intake, and anthropometric surveys) can be used to provide complementary insights into the nutritional profile of a population.^6^, ^38^, ^45^ The national burdens of micronutrient deficiencies are classified using biomarker surveys, but operational and analytical challenges can pose difficulties when interpreting results.^40^ Furthermore, some micronutrients that were indicated by this analysis to be of concern for dietary inadequacy are either not commonly assessed using biomarker analyses (e.g., riboflavin) or are challenging to measure using biomarkers, for example, owing to tight homeostatic control of concentrations in blood plasma/serum (e.g., zinc).^46^ Using dietary data to assess inadequacies in micronutrient intake can provide information to guide the design of policies centered around food‐based interventions. Additionally, dietary data can help to identify commonly consumed food items for future interventions^4^ and can be used to help identify populations at risk of deficiency when planning biomarker surveys and build context around which biomarker results can be interpreted. Dietary data will continue to play an integral role when describing the micronutrient profile of a population.

Mathematical modeling tools are valuable resources for providing policymakers with information to help guide nutrition investments and identify potential improvements to current nutrition policies. While many tools have been independently developed for various purposes, combining insight from multiple tool outputs could be valuable for informing policy and programs and may lead to increased uptake by end‐users.^47^ The policy relevance from our model outputs would be strengthened if it was possible to compare with other mathematical modeling tool outputs that describe other factors that influence decision making. This includes the cost‐effectiveness of current fortification policies and other micronutrient interventions,^48^ how to prioritize and bundle multiple micronutrient interventions to meet needs,^49^ and desirable food‐based alternatives to fortification to reduce inadequacy at the lowest cost.^50^ For many tools, mathematical models are dependent on up‐to‐date quality data, so while investment in advancing modeling techniques will remain important, parallel investment in survey design and data collection remains a priority.

## Strengths, limitations, and conclusions

This study had a number of strengths. When evaluating the micronutrient supply across the population, results were disaggregated by subpopulations to provide insight into which populations have the highest prevalence of dietary micronutrient inadequacy and which populations are likely to benefit most from the fortification of the selected vehicles. A novel adjustment of apparent intakes per AFE allowed for comparison between the apparent intake and micronutrient density approach when estimating dietary inadequacy. Additionally, the predictive model was developed to conduct the analysis across multiple fortification scenarios to predict the potential impact of different fortification scenarios using the selected vehicles. For some micronutrients, the analysis revealed fluctuations in the micronutrient supply from the overall diet across seasons, providing a more nuanced picture of the potential contribution of large‐scale food fortification.

There are several limitations in our study that should be noted. First, given that the food consumption data underpinning this study were at the household level, intrahousehold distribution of food between household members is unknown, and individual‐level estimates rely on a number of assumptions. Confirmation of the micronutrient status of any subpopulation of members within the household (e.g., PSC and women of reproductive age) will require data from individual‐level dietary and micronutrient biomarker assessments. For apparent intake, the assumptions adopted in this analysis focused exclusively on food distribution based on proportional energy requirements. However, the distribution of foods within the household is likely to be influenced by socioeconomic, gender, and cultural factors,^51^ and, depending on the context and household factors, certain demographic groups may be at greater risk of inadequate micronutrient intake due to inequitable food distribution.^52^ Alternative assumptions could be applied to explore scenarios that provided more or less energy than requirements for certain demographics to model the inequitable distribution of micronutrients between household members. While scenarios exploring additional research questions can be added to the current model framework, further research exploring the sensitivity of policy recommendations to the number of scenarios run is needed for clear model dissemination. Second, the micronutrient composition of food items, both naturally occurring and industrially processed, is assumed to be constant across all households, despite studies suggesting that the micronutrient composition of food items may vary due to numerous factors. In one study, Ulemu *et al*. found that vitamin A fortificant in oil deteriorates as the food product moves through the supply chain,^53^ suggesting that vitamin A concentration in fortified products is sensitive to the sun and air exposure and is likely to vary depending on the length of time since production and packaging/storage conditions. In addition, subnational spatial variation in the mineral micronutrient composition of staple crops has been reported in Malawi, likely due to soil properties and other environmental factors.^22^, ^54^ The current analysis assumed constant micronutrient composition of each food item, and further research identifying subnational variation in the micronutrient composition of food items could be integrated into our models. Third, our status quo scenario assumed that all oil, sugar, and wheat flour consumed by households is equally fortified. Market data of vitamin A contents of oil, sugar, and wheat flour collected from sentinel sites indicated that some samples were not fortified at all,^29^ despite our model applying the average from all sentinel sites equally. Finally, because we relied on HCES data to estimate the micronutrient intake and model the potential contributions of large‐scale food fortification, measurement error may have reduced the precision or accuracy of these estimates given the limitations of using HCES data (e.g., recall error, micronutrient loss during cooking, foods consumed away from home, fixed‐food item lists, and others).

In conclusion, this analysis demonstrated the use of HCES data and a novel mathematical modeling framework to estimate the coverage, potential contributions, and equity dimensions of large‐scale food fortification in Malawi. While large‐scale food fortification was predicted to have high potential contributions in reducing vitamin A inadequacies throughout most of Malawi, additional interventions are likely required to meet the needs of rural populations of low SEP. This information provides relevant insight for nutrition policy and programs when designing strategies to alleviate the overall burdens of micronutrient deficiencies, although further research is necessary to identify the optimal combination of interventions for future micronutrient investments.

## Author contributions

K.T., M.W., O.D., E.L.A., and E.J.M.J. set up the collaborative network. K.T., O.D., and E.J.M.J. designed the study. K.T., K.P.A., and B.L. cleaned and transformed the food consumption data. E.L.F. and L.S.D.L.R. compiled the food composition data matches. O.D. developed the food fortification scenarios. K.T., K.P.A., E.L.F., M.W., J.Y., O.D., A.A.K., S.P., E.L.A., and E.J.M.J. determined the adult female equivalent factor parameters. K.T. and B.C. defined the analysis for subpopulations. K.T. conducted the statistical analysis. K.T., K.P.A., and E.J.M.J. led the interpretation of the results and drafting of the manuscript. All authors critically reviewed and approved the final manuscript. K.T. is responsible for the integrity of the data analyzed.

## Competing interests

The authors declare no competing interests.

## Ethical standards disclosure

This study was approved by the London School of Hygiene and Tropical Medicine Observational Research Ethics Committee under the Micronutrient Action Policy Support (MAPS) project (ref. 21903; April 17, 2020).

## Peer review

The peer review history for this article is available at https://publons.com/publon/10.1111/nyas.14697.

## Supporting information


**Table S1**. Micronutrient composition for 100 g of food items from the IHS4 using food composition data from the Malawian (MWI),^1^ Kenyan (KEN),^2^ Lesothan (LSO),^3^ Mozambican (MOZ),^4^FAO West African (WAF),^5^ and the United Kingdom (UK)^6^ food composition tables.
**Table S2**. Fortifiable food equivalent factors for wheat flour in wheat flour products.
**Table S3**. Base parameters defining daily dietary micronutrient and energy requirements.
**Table S4**. Standard and nonstandard food consumption units recorded in Malawi's Fourth Integrated Household Survey.
**Table S5**. Prevalence of households exceeding the daily harmonized upper limit for vitamin A apparent intake across large‐scale food fortification scenarios by subpopulation.

Supplementary materials
**Figure S1**. Seasonality in the (A) nutrient density and (B) apparent intake of thiamine under the three fortification scenarios in relation to the inadequacy threshold (red dotted line) by socioeconomic position (lowest to highest) between urban and rural residences.
**Figure S2**. Seasonality in the (A) nutrient density and (B) apparent intake of riboflavin under the three fortification scenarios in relation to the inadequacy threshold (red dotted line) by socioeconomic position (lowest to highest) between urban and rural residences.
**Figure S3**. Seasonality in the (A) nutrient density and (B) apparent intake of niacin under the three fortification scenarios in relation to the inadequacy threshold (red dotted line) by socioeconomic position (lowest to highest) between urban and rural residences.
**Figure S4**. Seasonality in the (A) nutrient density and (B) apparent intake of vitamin B_6_ under the three fortification scenarios in relation to the inadequacy threshold (red dotted line) by socioeconomic position (lowest to highest) between urban and rural residences.
**Figure S5**. Seasonality in the (A) nutrient density and (B) apparent intake of folate under the three fortification scenarios by socioeconomic position (lowest to highest) between urban and rural residences.
**Figure S6**. Seasonality in the (A) nutrient density and (B) apparent intake of vitamin B_12_ under the three fortification scenarios in relation to the inadequacy threshold (red dotted line) by socioeconomic position (lowest to highest) between urban and rural residences.
**Figure S7**. Seasonality in the (A) nutrient density and (B) apparent intake of iron under the three fortification scenarios in relation to the inadequacy threshold (red dotted line) by socioeconomic position (lowest to highest) between urban and rural residences.
**Figure S8**. Histogram of apparent vitamin A intake per adult female equivalent in relation to the harmonized upper limit for daily vitamin A intake (dotted red line) under the three large‐scale food fortification scenarios.
