# Supplementary material for: Modeling food fortification contributions to micronutrient requirements in Malawi using Household Consumption and Expenditure Surveys
Source: Ann N Y Acad Sci. 2021 Sep 28;1508(1):105–22. doi: 10.1111/nyas.14697 (PMC9291765; doi:10.1111/nyas.14697)
Supplement: Supplementary file 1 — Table S1. Micronutrient composition for 100 g of food items from the IHS4 using food composition data from the Malawian (MWI),1 Kenyan (KEN),2 Lesothan (LSO),3 Mozambican (MOZ),4FAO West African (WAF),5 and the United Kingdom (UK)6 food composition tables. Table S2. Fortifiable food equivalent factors for wheat flour in wheat flour products. Table S3. Base parameters defining daily dietary micronutrient and energy requirements. Table S4. Standard and nonstandard food consumption units recorded in Malawi's Fourth Integrated Household Survey. Table S5. Prevalence of households exceeding the daily harmonized upper limit for vitamin A apparent intake across large‐scale food fortification scenarios by subpopulation. [file NYAS-1508-105-s001.docx]

Modeling food fortification contributions to micronutrient requirements in Malawi using Household Consumption and Expenditure Surveys

**SUPPLEMENTARY MATERIAL**

Table of Contents

[Description of data cleaning and transformation procedures 2](#_Toc82521560)

[Description of adult female equivalent factor calculation 4](#_Toc82521561)

[References 6](#_Toc82521562)

# Description of data cleaning and transformation procedures

**Conversion of non-standard units**

IHS4 food consumption data measured the quantity consumed of each food item using a list of standard and non-standard units (Table S4). All units for all food items were converted into kilograms using regionally specific non-standard unit to kilogram conversion factors made available by the World Bank’s Living Standards and Measurement Study. For food item units with missing conversion factors, kilogram conversion factors were estimated equally across all regions either using the IHS4 “Household food consumption photo aid” or by calculating the food quantity’s mass using volumes of non-standard units purchased from local markets in Malawi. Food item units that were reported as “Other (specify)” which were similar to listed non-standard units were reported as their listed equivalent, and converted into kilograms as accordingly. Food item units that were reported as “Other (specify)” without equivalent listed non-standard units were disregarded due to the absence of an appropriate conversion factor.

**Non-edible portions of food**

We adjusted for non-edible portions of food weight included in the seven-day household recall (e.g. potato peels, maize husks, maize cobs). Proportion factors of non-edible portions for certain food items were estimated using refuse percentages from matched items of the 2019 FAO West African Food Composition Tables.^5^

**Food composition table match**

Each IHS4 food item was screened to identify a potential corresponding food item in the 2019 Malawi Food Composition Table (MAFOODS).^1^ The screening process was done manually. We gathered information regarding each IHS4 food item, corresponding food items in MAFOODS, and classified each food item by the type of match through four categories: ‘exact match’, ‘partial match’, ‘multiple matches’, or ‘no match’.

Food items that were identified as ‘exact match’ had a one-to-one perfect match, that is identical food items and method of preparation.

Food items identified as ‘partial match’ were similar to the food consumed in MAFOODS but not perfect (e.g. “brown beans”, as reported in IHS4, was matched to “kidney beans” in MAFOODS). We made adjustments to the food composition to ensure consistency between the recalled food item processing and the match item on the food composition table (e.g. IHS4 “dried fish” matched to a fish item which adjusted composition data to reflect drying process).

Food items that were identified as ‘multiple matches’ were evaluated to identify a single, most representative item. Some IHS4 food items were listed as broad food categories (e.g. IHS4’s “Small Fresh Fish”). To find the equivalent item in MAFOODS, the median of multiple MAFOODS items was calculated to account for variability in the food composition of different varieties of the same food. All the decisions were informed by published data and experts’ opinion and recorded in the spreadsheet.

Finally, ‘no match’ were defined as IHS4 food items with either no equivalent in MAFOODS or had equivalents but with missing values for one or more nutrients. In this case, data from other food composition tables were used. Food composition tables were selected based on geographic proximity to Malawi and quality of the data, being, in order of preference, 2018 Kenya Food Composition Table^2^, 2019 Food Composition Table for Western Africa compiled by the Food and Agriculture Organization of the United Nations ^5^, 2006 Lesotho Food Composition Table^3^ and the 2011 Mozambique Food Composition Tables.^4^ For globally traded food items and items with missing composition data from all neighboring country food composition tables, food items were matched to corresponding food items from McCance and Widdowson’s food composition table for the United Kingdom. ^6^

After food matching, all missing values were identified, and nutrient values and metadata were imputed from an equivalent food item using the above-mentioned food composition tables. The script used to create the final match and metadata spreadsheet used in this study is available upon request.

In total, 65% of the food items were matched to a food item reported in MAFOODS, while the rest of the items were sourced from other food composition tables. These matches were reviewed and agreed upon between two researchers. Any disagreements between the two co-authors about whether to include a publication were resolved through discussion.

Many food items were recalled by households in their raw market purchased variety (e.g. grams of dry spaghetti, bunches of uncooked green vegetables, buckets of uncooked potatoes) without providing any information on processing and cooking once obtained by the household. We choose to analyze the raw variety of these food items without making any assumptions about changes in yield or addition/loss of micronutrients during the cooking process as processing of food items may vary substantially between households. These household micronutrient supply estimates are expected to be greater than the total household micronutrient intake as some micronutrients are expected to be lost during the cooking process.

**Management of outliers**

Food item consumption quantities that exceed a pre-specified threshold value will be corrected and capped. Total consumption quantity of individual food items per capita per day demonstrates a non-parametric distribution with a right skew. Each food items’ consumption quantity distribution will be normalized through logarithmic transformation and extreme consumption quantity values, or values greater than five standard deviations above the mean of the logarithmically transformed consumption quantities, will be defined as outliers. Outliers will be replaced with the population median consumption quantity among consumers for each food item.

# Description of adult female equivalent factor calculation

For the apparent intake approach, household supply of each micronutrient will be divided by the number of adult female equivalents within the household. The subsequent steps will outline the process necessary to calculate the total number of adult female equivalents (AFE) for each household, where AFEs are based on the energy requirements of each member of the household. The following data and assumptions will be combined to estimate each household members’ energy requirements as defined by Weisell & Dop.^17^ All AFE base parameters, data sources, and assumptions are presented in Table S3.

**Base energy requirements**

Base energy expenditure for all household members will be based on the Human Energy Requirements recommendations from the Joint FAO/WHO/UNU Expert Consultation.^9^ Data describing individual household members’ age and sex is collected as part of the IHS4-Module B “Household Roster.” Household member’s sex is described in question “B03- Sex” and age is described in question “B06_3- How old is [NAME]?”.

###

The body weight of individual household members was not collected as part of IHS4. For females, we used data from Malawi’s Demographic and Health Survey (DHS) from 2015/16 ^10^ describing non-pregnant females (n=7180) where the average weight across the entire country was 55.9 kg. Weights of males were not collected in the 2015/16 DHS, so we assumed energy requirements necessary for males with a weight of 65 kg as the adult male standard. This analysis assumed that all adult household members expend “moderate physical activity levels” or a factor of 1.6 basal metabolic rates.

**Additional energy requirements for pregnancy**

Data describing the pregnancy status of individual household members is collected as part of the IHS4-Module D “Health.” Pregnancy status of household members is described in question “D04- During the past 2 weeks have you suffered from an illness or injury? (Y/N)” and “D05- What was the illness or injury?” where the categorical response “28” indicates “Pregnancy.” Pregnant women will have additional energy added to their base energy requirements, where pregnant women will require an additional 300 kcal per day.^11^

**Additional energy requirements for lactation**

According to Malawi’s 2015/16 DHS, breastfeeding adherence was high, where 89% of infants between ages 12-23 months continued to be breastfed.^13^ We assumed that all households with children below 2 years old will have a lactating mother in accordance with the Worth Health Organization recommendations for breastfeeding ^14^. This is expected to be an overestimation of the total number of lactating women since there is likely to be variation in the degree of adherence to international breastfeeding guidelines, however with high breastfeeding adherence in Malawi we expected this to make little difference in estimates of apparent intake. Lactating women will have additional energy added to their base energy requirements, where lactating women will require an additional 500 kcal per day.^12^

**Energy contributions from breastmilk for infants under 2 years old**

For infants under 2 years old, energy contributions from breastmilk were subtracted from their daily energy requirements to represent their total energy requirements from complementary foods. The total energy intake from breastmilk was age stratified for infants between 0-2 month, 3-5 months, 6-8 months, 9-11 months, and 12-23 months, which is consistent with the age stratifications from a systematic review that summarized breastmilk intake studies from developing countries. ^14^ For children under 6 months of age, Malawi’s 2015/16 DHS data was used to provide insight into the proportion of children under 6 months that were exclusively breastfeeding, where the crude approach would be to assume that all children under 6 months were exclusively breastfeeding and didn’t consume any of the family meal. Only 60% of children under 6 months in Malawi were exclusively breastfeeding, where children between 0-2 months had high exclusive breastfeeding adherence (>90%) and children between 3-6 months had lower adherence (~40%). With this, in the AFE estimates used for the HCES model, we assumed children under 2 were exclusively breastfed and did not take part in the family meal, where children aged 3-6 months did partially take part in the family meal.

# References

1. Van Graan A., J. Chetty, M. Jumat, *et al.* 2019. “*Malawian Food Composition Table*.” Lilongwe.

2. Food and Agriculture Organization of the United Nations & Government of Kenya. 2018. “*Kenya Food Composition Tables*.” Nairobi.

3. Lephole M.M., M.C. Khaketla & M.E. Monoto. 2006. “*Composition of Lesotho Foods*.” Maseru.

4. Korkalo L., H. Hauta-alus & M. Mutanen. 2011. “*Food Composition Tables for Mozambique*.” Maputo.

5. Vincent A., F. Grande, E. Compaoré, *et al.* 2020. “*FAO/INFOODS Food Composition Table for Western Africa*.” Rome.

6. McCance R. & E. Widdowson. 2020. “*McCance and Widdowson’s The Composition of Foods Integrated Dataset*.” London.

7. Engle-Stone R., M. Nankap, A.O. Ndjebayi, *et al.* 2014. Simulations based on representative 24-h recall data predict region-specific differences in adequacy of vitamin a intake among Cameroonian women and young children following large-scale fortification of vegetable oil and other potential food vehicles. *J. Nutr.* **144**: 1826–34.

8. National Statistical Office & The World Bank. 2017. “*Fourth Integrated Household Survey of Malawi*.” Lilongwe.

9. FAO/WHO/UNU. 2004. Human energy requirements. Report of a Joint FAO/WHO/UNU Expert Consultation: Rome, 17–24 October 2001. *AO food Nutr. Tech. Rep. Ser.*

10. National Statistics Office (NSO). 2017. “*Malawi Demographic and Health Survey 2015-16*.” Zomba.

11. Kominiarek M.A. & P. Rajan. 2016. Nutrition Recommendations in Pregnancy and Lactation. *Med. Clin. North Am.* **100**: 1199–1215.

12. US Centers for Disease Control and Prevention. Accessed October 8, 2020. https://www.cdc.gov/breastfeeding/breastfeeding-special-circumstances/diet-and-micronutrients/maternal-diet.html.

13. Walters C.N., H. Rakotomanana, J.J. Komakech, *et al.* 2019. Maternal determinants of optimal breastfeeding and complementary feeding and their association with child undernutrition in Malawi (2015-2016). *BMC Public Health* **19**: 1503.

14. WHO Programme of Nutrition. 1998. “*Complementary feeding of young children in developing countries: a review of current scientific knowledge*.” Geneva.

15. Allen L.H., A.L. Carriquiry & S.P. Murphy. 2020. Proposed Harmonized Nutrient Reference Values for Populations. *Adv. Nutr.* **11**: 469–483.

16. Allen, L., de Benoist, B., Dary, O. & Hurrell R. 2006. “*Guidelines on food fortification with micronutrients*.” Geneva.

17. Weisell R. & M.C. Dop. 2012. The adult male equivalent concept and its application to Household Consumption and Expenditures Surveys (HCES). *Food Nutr. Bull.* **33**: S157-62.
