# Supplementary material for: Modeling food fortification contributions to micronutrient requirements in Malawi using Household Consumption and Expenditure Surveys
Source: Ann N Y Acad Sci. 2021 Sep 28;1508(1):105–22. doi: 10.1111/nyas.14697 (PMC9291765; doi:10.1111/nyas.14697)
Supplement: Supplementary file 2 — Supplementary materials Figure S1. Seasonality in the (A) nutrient density and (B) apparent intake of thiamine under the three fortification scenarios in relation to the inadequacy threshold (red dotted line) by socioeconomic position (lowest to highest) between urban and rural residences. Figure S2. Seasonality in the (A) nutrient density and (B) apparent intake of riboflavin under the three fortification scenarios in relation to the inadequacy threshold (red dotted line) by socioeconomic position (lowest to highest) between urban and rural residences. Figure S3. Seasonality in the (A) nutrient density and (B) apparent intake of niacin under the three fortification scenarios in relation to the inadequacy threshold (red dotted line) by socioeconomic position (lowest to highest) between urban and rural residences. Figure S4. Seasonality in the (A) nutrient density and (B) apparent intake of vitamin B6 under the three fortification scenarios in relation to the inadequacy threshold (red dotted line) by socioeconomic position (lowest to highest) between urban and rural residences. Figure S5. Seasonality in the (A) nutrient density and (B) apparent intake of folate under the three fortification scenarios by socioeconomic position (lowest to highest) between urban and rural residences. Figure S6. Seasonality in the (A) nutrient density and (B) apparent intake of vitamin B12 under the three fortification scenarios in relation to the inadequacy threshold (red dotted line) by socioeconomic position (lowest to highest) between urban and rural residences. Figure S7. Seasonality in the (A) nutrient density and (B) apparent intake of iron under the three fortification scenarios in relation to the inadequacy threshold (red dotted line) by socioeconomic position (lowest to highest) between urban and rural residences. Figure S8. Histogram of apparent vitamin A intake per adult female equivalent in relation to the harmonized upper limit for daily vitamin A intake (dotted red line) u [file NYAS-1508-105-s002.docx]

Modeling food fortification contributions to micronutrient requirements in Malawi using Household Consumption and Expenditure Surveys

**Supplementary Tables and Figures**

Table of Contents

[Model Parameters 2](#_Toc82521439)

[Seasonality plots for additional micronutrients 10](#_Toc82521440)

[Prevalence of apparent vitamin A intake above the Harmonized-Upper Limit 18](#_Toc82521441)

[References 20](#_Toc82521442)

# Model Parameters

| ***Table S1.*** *Micronutrient composition for 100 grams of food items from the IHS4 using food composition data from the Malawian (MWI)*^1^*, Kenyan (KEN)*^2^*, Lesothan (LSO)*^3^*, Mozambican (MOZ)*^4^*, FAO West African (WAF)*^5^ *and United Kingdom (UK)*^6^ *food composition tables.* | | | | | | | | | | | | | | | | | | |  |  |
| --- | --- | --- | --- | --- | --- | --- | --- | --- | --- | --- | --- | --- | --- | --- | --- | --- | --- | --- | --- | --- |
| **Food item** | **VitA**  *μg RAE* | **Ref** | **Thia**  *mg* | **Ref** | **Ribo**  *mg* | **Ref** | **Niac**  *mg* | **Ref** | **VitB6**  *mg* | **Ref** | **Fol**  *μg* | **Ref** | **VitB12**  *μg* | **Ref** | **Iron**  *mg* | **Ref** | **Zinc**  *mg* | **Ref** | **Energy**  *kcal* | **Ref** |
| Maize ufa mgaiwa (normal flour) | 0 | MWI | 0.50 | MWI | 0.12 | MWI | 1.4 | MWI | 0.37 | MWI | 25 | MWI | 0 | MWI | 3.8 | MWI | 1.7 | MWI | 374 | MWI |
| Maiza ufa refined (refined flour) | 0 | MWI | 0.13 | MWI | 0.04 | MWI | 0.8 | MWI | 0.08 | MWI | 10 | MWI | 0 | MWI | 1.0 | MWI | 0.5 | MWI | 367 | MWI |
| Maize ufa madeya (bran flour) | 2 | MWI | 0.25 | MWI | 0.11 | MWI | 2.4 | MWI | 0.22 | MWI | 18 | MWI | 0 | MWI | 2.7 | MWI | 3.0 | MWI | 373 | MWI |
| Maize grain | 0 | KEN | 0.35 | MWI | 0.10 | MWI | 2.1 | MWI | 0.20 | MWI | 26 | MWI | 0 | MWI | 1.4 | MWI | 1.6 | MWI | 370 | MWI |
| Green maize | 0 | MWI | 0.08 | MWI | 0.03 | MWI | 0.6 | MWI | 0.05 | MWI | 7 | MWI | 0 | MWI | 1.1 | MWI | 0.8 | MWI | 133 | MWI |
| Rice | 0 | MWI | 0.15 | MWI | 0.03 | MWI | 0.2 | MWI | 0.16 | MWI | 8 | MWI | 0 | MWI | 0.4 | MWI | 1.6 | MWI | 348 | MWI |
| Finger millet | 0 | MWI | 0.31 | MWI | 0.13 | MWI | 1.8 | MWI | 0.75 | MWI | 30 | MWI | 0 | MWI | 10.1 | MWI | 1.8 | MWI | 378 | MWI |
| Sorghum | 1 | MWI | 0.35 | MWI | 0.16 | MWI | 3.2 | MWI | 0.25 | MWI | 29 | MWI | 0 | MWI | 12.6 | MWI | 1.8 | MWI | 358 | MWI |
| Pearl millet | 0 | WAF | 0.20 | WAF | 0.20 | WAF | 1.9 | WAF | 0.32 | WAF | 47 | WAF | 0 | WAF | 5.4 | WAF | 2.4 | WAF | 370 | WAF |
| Wheat flour | 0 | WAF | 0.21 | WAF | 0.09 | WAF | 2.4 | WAF | 0.45 | WAF | 240 | WAF | 0 | WAF | 2.0 | WAF | 0.5 | WAF | 352 | WAF |
| Bread | 48 | MWI | 0.24 | MWI | 0.17 | MWI | 1.8 | MWI | 0.16 | MWI | 38 | MWI | 0.1 | MWI | 2.1 | MWI | 0.6 | MWI | 304 | MWI |
| Buns, scones | 32 | MWI | 0.11 | MWI | 0.17 | MWI | 0.3 | MWI | 0.04 | MWI | 13 | MWI | 0.3 | MWI | 1.8 | MWI | 0.4 | MWI | 224 | MWI |
| Biscuits | 40 | KEN | 0.09 | KEN | 0.03 | KEN | 0.7 | LSO | 0.05 | KEN | 12 | KEN | 0.1 | KEN | 1.4 | KEN | 0.6 | KEN | 460 | KEN |
| Spaghetti, macaroni, pasta | 0 | KEN | 0.07 | KEN | 0.06 | KEN | 1.1 | WAF | 0.13 | KEN | 18 | KEN | 0 | KEN | 1.0 | KEN | 0.6 | KEN | 354 | KEN |
| Breakfast cereal | 5 | KEN | 0.13 | KEN | 0.06 | KEN | 1.5 | LSO | 0.03 | KEN | 5 | KEN | 0 | WAF | 1.5 | KEN | 0.4 | KEN | 340 | KEN |
| Cassava tubers | 1 | MWI | 0.04 | MWI | 0.05 | MWI | 0.7 | MWI | 0.09 | MWI | 24 | MWI | 0 | MWI | 0.4 | MWI | 0.4 | MWI | 160 | MWI |
| Cassava flour | 0 | WAF | 0.07 | MWI | 0.11 | MWI | 1.2 | MWI | 0.17 | MWI | 46 | MWI | 0 | MWI | 0.5 | MWI | 0.7 | WAF | 348 | MWI |
| White sweet potato | 2 | MWI | 0.07 | MWI | 0.03 | MWI | 0.4 | MWI | 0.20 | MWI | 38 | MWI | 0 | MWI | 0.2 | MWI | 0.2 | MWI | 89 | MWI |
| Orange sweet potato | 926 | MWI | 0.03 | MWI | 0.05 | MWI | 1.0 | MWI | 0.20 | MWI | 11 | MWI | 0 | MWI | 0.5 | MWI | 0.5 | MWI | 81 | MWI |
| Irish potato | 0 | KEN | 0.16 | MWI | 0.01 | MWI | 1.4 | MWI | 0.21 | MWI | 3 | MWI | 0 | MWI | 0.7 | MWI | 0.3 | MWI | 68 | MWI |
| Potato crisps | 0 | KEN | 0.19 | MWI | 0.01 | MWI | 2.0 | MWI | 0.30 | MWI | 3 | MWI | 0 | MWI | 1.0 | MWI | 0.4 | MWI | 264 | MWI |
| Plantain | 38 | MWI | 0.05 | MWI | 0.04 | MWI | 0.5 | MWI | 0.20 | MWI | 13 | MWI | 0 | MWI | 0.8 | MWI | 0.1 | MWI | 124 | MWI |
| Cocoyam | 0 | WAF | 0.10 | MWI | 0.03 | MWI | 0.8 | MWI | 0.24 | MWI | 22 | MWI | 0 | MWI | 0.6 | MWI | 1.4 | MWI | 137 | MWI |
| Beans, white | 0 | WAF | 0.89 | WAF | 0.11 | WAF | 1.5 | WAF | 0.42 | WAF | 410 | WAF | 0 | WAF | 8.8 | WAF | 3.2 | WAF | 320 | WAF |
| Beans, brown | 0 | MWI | 0.54 | MWI | 0.22 | MWI | 2.1 | MWI | 0.40 | MWI | 397 | MWI | 0 | MWI | 7.7 | MWI | 3.3 | MWI | 343 | MWI |
| Pigeon pea | 8 | MWI | 0.61 | MWI | 0.18 | MWI | 2.7 | MWI | 0.27 | MWI | 256 | MWI | 0 | MWI | 6.1 | MWI | 2.2 | MWI | 364 | MWI |
| Groundnut flour | 0 | MWI | 0.86 | MWI | 0.14 | MWI | 9.5 | MWI | 0.58 | MWI | 108 | MWI | 0 | MWI | 3.8 | MWI | 2.8 | MWI | 583 | MWI |
| Soyabean flour | 1 | MWI | 0.70 | MWI | 0.08 | MWI | 2.0 | MWI | 0.82 | MWI | 376 | MWI | 0 | MWI | 9.5 | MWI | 3.5 | MWI | 458 | MWI |
| Ground bean | 1 | WAF | 0.77 | WAF | 0.19 | WAF | 2.3 | WAF | 0.34 | WAF | 480 | WAF | 0 | WAF | 10.0 | WAF | 1.1 | WAF | 322 | WAF |
| Cowpea | 3 | MWI | 0.72 | MWI | 0.15 | MWI | 3.1 | MWI | 0.36 | MWI | 421 | MWI | 0 | MWI | 5.4 | MWI | 2.8 | MWI | 349 | MWI |
| Macadamia nuts | 0 | KEN | 0.27 | KEN | 0.10 | KEN | 2.0 | UK | 0.28 | UK | 0 | UK | 0 | KEN | 2.2 | KEN | 1.3 | KEN | 696 | KEN |
| Groundnut (shelled) | 0 | MWI | 0.87 | MWI | 0.14 | MWI | 15.5 | MWI | 0.59 | MWI | 110 | MWI | 0 | MWI | 1.9 | MWI | 2.5 | MWI | 597 | MWI |
| Groundnut (unshelled) | 0 | MWI | 0.87 | MWI | 0.14 | MWI | 15.5 | MWI | 0.59 | MWI | 110 | MWI | 0 | MWI | 1.9 | MWI | 2.5 | MWI | 597 | MWI |
| Groundnut fresh (unshelled) | 0 | MWI | 0.47 | MWI | 0.08 | MWI | 8.4 | MWI | 0.32 | MWI | 60 | MWI | 0 | MWI | 2.1 | MWI | 1.4 | MWI | 324 | MWI |
| Soya | 1 | MWI | 0.71 | MWI | 0.08 | MWI | 2.0 | MWI | 0.83 | MWI | 378 | MWI | 0 | MWI | 17.3 | MWI | 3.9 | MWI | 432 | MWI |
| Onion | 0 | MWI | 0.03 | MWI | 0.01 | MWI | 0.1 | MWI | 0.12 | MWI | 2 | MWI | 0 | MWI | 0.5 | MWI | 0.4 | MWI | 43 | MWI |
| Cabbage | 0 | KEN | 0.04 | MWI | 0.02 | MWI | 0.3 | MWI | 0.08 | MWI | 15 | MWI | 0 | MWI | 0.9 | MWI | 0.3 | MWI | 32 | MWI |
| Tanaposi/rape | 579 | MWI | 0.07 | MWI | 0.10 | MWI | 0.6 | MWI | 0.26 | MWI | 194 | MWI | 0 | MWI | 2.8 | MWI | 0.4 | MWI | 37 | MWI |
| Nkhwani | 126 | MWI | 0.05 | MWI | 0.07 | MWI | 0.5 | MWI | 0.11 | MWI | 32 | MWI | 0 | MWI | 5.9 | MWI | 0.4 | MWI | 27 | MWI |
| Chinese cabbage | 27 | KEN | 0.02 | MWI | 0.02 | MWI | 0.5 | MWI | 0.05 | MWI | 13 | MWI | 0 | MWI | 0.3 | MWI | 0.6 | MWI | 19 | MWI |
| Other cultivated green leafy veg. | 277 | MWI | 0.13 | MWI | 0.33 | MWI | 0.9 | MWI | 0.25 | MWI | 118 | MWI | 0 | MWI | 5.8 | MWI | 0.7 | MWI | 52 | MWI |
| Gathered wild green leaves | 303 | MWI | 0.16 | MWI | 0.42 | MWI | 1.6 | MWI | 0.41 | MWI | 286 | MWI | 0 | MWI | 21.9 | MWI | 1.6 | MWI | 62 | MWI |
| Tomato | 42 | MWI | 0.04 | MWI | 0.02 | MWI | 0.6 | MWI | 0.08 | MWI | 15 | MWI | 0 | MWI | 0.5 | MWI | 0.1 | MWI | 26 | MWI |
| Cucumber | 0 | KEN | 0.03 | KEN | 0.02 | KEN | 0.2 | WAF | 0.03 | WAF | 5 | KEN | 0 | KEN | 0.7 | KEN | 0.2 | WAF | 11 | KEN |
| Pumpkin | 189 | MWI | 0.07 | MWI | 0.04 | MWI | 0.7 | MWI | 0.15 | MWI | 9 | MWI | 0 | MWI | 2.0 | MWI | 0.2 | MWI | 60 | MWI |
| Okra/Therere | 41 | MWI | 0.06 | MWI | 0.13 | MWI | 1.1 | MWI | 0.35 | MWI | 138 | MWI | 0 | MWI | 1.3 | MWI | 0.9 | MWI | 61 | MWI |
| Tinned vegetables | 16 | KEN | 0.12 | KEN | 0.03 | KEN | 1.0 | UK | 0.11 | UK | 23 | UK | 0 | KEN | 0.8 | KEN | 0.1 | KEN | 19 | KEN |
| Mushroom | 2 | MWI | 0.10 | MWI | 0.27 | MWI | 3.8 | MWI | 0.08 | MWI | 29 | MWI | 0 | MWI | 0.2 | MWI | 0.6 | MWI | 30 | MWI |
| Eggs | 67 | MWI | 0.13 | MWI | 0.40 | MWI | 0.1 | MWI | 0.04 | MWI | 46 | MWI | 1.9 | MWI | 1.8 | MWI | 1.2 | MWI | 148 | MWI |
| Beef | 7 | KEN | 0.06 | MWI | 0.20 | MWI | 6.5 | MWI | 0.24 | MWI | 7 | MWI | 1.5 | MWI | 7.5 | MWI | 1.8 | MWI | 95 | MWI |
| Goat | 0 | MWI | 0.18 | MWI | 0.29 | MWI | 6.1 | MWI | 0.40 | MWI | 5 | MWI | 1.1 | MWI | 2.4 | MWI | 3.5 | MWI | 165 | MWI |
| Pork | 0 | MWI | 0.72 | MWI | 0.22 | MWI | 3.8 | MWI | 0.32 | MWI | 2 | MWI | 0.8 | MWI | 1.4 | MWI | 3.6 | MWI | 265 | MWI |
| Mutton | 10 | MWI | 0.13 | MWI | 0.19 | MWI | 3.5 | MWI | 0.40 | MWI | 2 | MWI | 2.9 | MWI | 2.1 | MWI | 3.3 | MWI | 257 | MWI |
| Chicken | 7 | MWI | 0.09 | MWI | 0.06 | MWI | 3.7 | MWI | 0.27 | MWI | 2 | MWI | 0.2 | MWI | 1.0 | MWI | 1.6 | MWI | 129 | MWI |
| Other poultry | 11 | MWI | 0.23 | MWI | 0.24 | MWI | 8.0 | MWI | 0.51 | MWI | 4 | MWI | 0.6 | MWI | 2.2 | MWI | 2.6 | MWI | 121 | MWI |
| Small animal (rabbit, mice etc.) | 10 | MWI | 0.11 | MWI | 0.15 | MWI | 9.5 | MWI | 0.57 | MWI | 5 | MWI | 10.0 | MWI | 1.2 | MWI | 1.7 | MWI | 130 | MWI |
| Insect (e.g. termite) | 346 | KEN | 0.20 | KEN | 0.70 | KEN | 1.6 | WAF | 0.40 | WAF | 157 | WAF | 0 | KEN | 117 | KEN | 0.6 | WAF | 194 | MWI |
| Tinned meat or fish | 8 | WAF | 0.09 | WAF | 0.11 | WAF | 1.6 | WAF | 0.12 | WAF | 4 | WAF | 1.0 | WAF | 0.9 | WAF | 1.6 | WAF | 234 | WAF |
| Fish soup/sauce | 23 | MWI | 0.04 | MWI | 0.01 | MWI | 0.6 | MWI | 0.07 | MWI | 9 | MWI | 0 | MWI | 6.6 | MWI | 2.0 | MWI | 44 | MWI |
| Chicken pieces | 7 | MWI | 0.09 | MWI | 0.06 | MWI | 3.7 | MWI | 0.27 | MWI | 2 | MWI | 0.2 | MWI | 1.0 | MWI | 1.6 | MWI | 129 | MWI |
| Sun dried fish (large) | 44 | MWI | 0.24 | MWI | 0.22 | MWI | 4.2 | MWI | 0.87 | MWI | 21 | MWI | 12.0 | MWI | 23.6 | MWI | 4.6 | MWI | 375 | MWI |
| Sun dried fish (medium) | 44 | MWI | 0.24 | MWI | 0.22 | MWI | 4.2 | MWI | 0.87 | MWI | 21 | MWI | 12.0 | MWI | 23.6 | MWI | 4.6 | MWI | 375 | MWI |
| Sun dried fish (small) | 107 | MWI | 0.03 | MWI | 0.36 | MWI | 19.5 | MWI | 1.79 | MWI | 18 | MWI | 21.1 | MWI | 13.3 | MWI | 13.5 | MWI | 346 | MWI |
| Fresh fish (large) | 10 | MWI | 0.06 | MWI | 0.09 | MWI | 3.1 | MWI | 0.26 | MWI | 14 | MWI | 2.5 | MWI | 2.9 | MWI | 4.3 | MWI | 99 | MWI |
| Fresh fish (medium) | 10 | MWI | 0.06 | MWI | 0.09 | MWI | 3.1 | MWI | 0.26 | MWI | 14 | MWI | 2.5 | MWI | 2.9 | MWI | 4.3 | MWI | 99 | MWI |
| Fresh fish (small) | 12 | WAF | 0.03 | WAF | 0.12 | WAF | 2.6 | WAF | 0.24 | WAF | 15 | UK | 1.9 | KEN | 3.8 | WAF | 1.2 | UK | 126 | MWI |
| Smoked fish (large) | 414 | MWI | 0.29 | MWI | 0.32 | MWI | 5.6 | MWI | 0.88 | MWI | 17 | MWI | 9.8 | MWI | 100.7 | MWI | 4.5 | MWI | 394 | MWI |
| Smoked fish (medium) | 414 | MWI | 0.29 | MWI | 0.32 | MWI | 5.6 | MWI | 0.88 | MWI | 17 | MWI | 9.8 | MWI | 100.7 | MWI | 4.5 | MWI | 394 | MWI |
| Smoked fish (small) | 107 | MWI | 0.09 | MWI | 0.13 | MWI | 13.4 | MWI | 0.43 | MWI | 25 | MWI | 2.7 | MWI | 10.7 | MWI | 16.4 | MWI | 194 | MWI |
| Mango | 123 | MWI | 0.05 | MWI | 0.03 | MWI | 0.1 | MWI | 0.06 | MWI | 34 | MWI | 0 | MWI | 0.3 | MWI | 0.1 | MWI | 66 | MWI |
| Banana | 4 | MWI | 0.04 | MWI | 0.04 | MWI | 0.6 | MWI | 0.36 | MWI | 20 | MWI | 0 | MWI | 0.3 | MWI | 0.2 | MWI | 109 | MWI |
| Citrus (naartje, orange etc) | 5 | WAF | 0.06 | MWI | 0.02 | MWI | 0.3 | MWI | 0.04 | MWI | 24 | MWI | 0 | MWI | 0.3 | MWI | 0.2 | MWI | 51 | MWI |
| Pineapple | 6 | WAF | 0.08 | MWI | 0.02 | MWI | 0.3 | MWI | 0.06 | MWI | 16 | MWI | 0 | MWI | 0.3 | MWI | 0 | MWI | 58 | MWI |
| Papaya | 16 | MWI | 0.03 | MWI | 0.01 | MWI | 0.3 | MWI | 0.01 | MWI | 15 | MWI | 0 | MWI | 0.4 | MWI | 0.1 | MWI | 48 | MWI |
| Guava | 5 | MWI | 0.05 | MWI | 0.02 | MWI | 1.2 | MWI | 0.79 | MWI | 19 | MWI | 0 | MWI | 0.4 | MWI | 0.2 | MWI | 71 | MWI |
| Avocado | 6 | MWI | 0.06 | MWI | 0.14 | MWI | 1.7 | MWI | 0.32 | MWI | 33 | MWI | 0 | MWI | 0.7 | MWI | 0.3 | MWI | 152 | MWI |
| Wild fruit (masau, malimbe, etc.) | 15 | MWI | 0.13 | MWI | 0.09 | MWI | 0.9 | MWI | 0.09 | MWI | 20 | MWI | 0 | MWI | 3.0 | MWI | 0.8 | MWI | 167 | MWI |
| Apple | 1 | WAF | 0.02 | MWI | 0.06 | MWI | 0.2 | MWI | 0.03 | MWI | 1 | MWI | 0 | MWI | 0.3 | MWI | 0.1 | MWI | 63 | MWI |
| Fresh milk | 44 | MWI | 0.02 | MWI | 0.16 | MWI | 0.1 | MWI | 0.04 | MWI | 5 | MWI | 0.4 | MWI | 0.1 | MWI | 0.4 | MWI | 67 | MWI |
| Powdered milk | 329 | WAF | 0.3 | WAF | 1.20 | WAF | 0.8 | WAF | 0.25 | WAF | 37 | WAF | 1.8 | WAF | 0.8 | WAF | 3.3 | WAF | 493 | WAF |
| Margarine- Blue band | 850 | MWI | 1.3 | MWI | 1.70 | MWI | 18.0 | MWI | 2.00 | MWI | 200 | MWI | 1.0 | MWI | 0 | MWI | 0 | MWI | 724 | MWI |
| Butter | 845 | KEN | 0 | KEN | 0.05 | KEN | 1.1 | WAF | 0 | WAF | 5 | KEN | 0 | KEN | 0 | KEN | 0.1 | KEN | 735 | KEN |
| Chambiko (soured milk) | 17 | KEN | 0.02 | LSO | 0.15 | LSO | 0 | WAF | 0.02 | WAF | 7 | LSO | 0.2 | LSO | 0.1 | LSO | 0.6 | LSO | 65 | LSO |
| Yoghurt | 34 | KEN | 0.1 | KEN | 0.20 | KEN | 0.1 | WAF | 0.05 | WAF | 8 | KEN | 0.2 | KEN | 0.2 | WAF | 0.3 | KEN | 85 | KEN |
| Cheese | 68 | KEN | 0.04 | KEN | 0.32 | KEN | 0.7 | WAF | 0.04 | WAF | 21 | KEN | 0.9 | KEN | 0.3 | KEN | 1.1 | KEN | 184 | KEN |
| Sugar | 0 | KEN | 0 | KEN | 0 | KEN | 0 | WAF | 0 | WAF | 0 | KEN | 0 | KENI | 0 | KEN | 0 | KEN | 400 | KEN |
| Sugar cane | 0 | MWI | 0.03 | KEN | 0.04 | KEN | 0.1 | UK | 0 | MWI | 0 | KEN | 0 | MWI | 0.6 | MWI | 0.1 | KEN | 40 | MWI |
| Cooking oil | 0 | WAF | 0 | WAF | 0 | WAF | 0 | WAF | 0 | WAF | 0 | WAF | 0 | WAF | 0.1 | WAF | 0 | WAF | 900 | WAF |
| Salt | 0 | KEN | 0 | KEN | 0 | KEN | 0 | UK | 0 | WAF | 0 | KEN | 0 | KEN | 0.1 | KEN | 0.1 | KEN | 0 | KEN |
| Spices | 55 | KEN | 0.25 | KEN | 0.45 | KEN | 4.9 | UK | 0.21 | WAF | 36 | KEN | 0 | KEN | 35.5 | KEN | 3.7 | UK | 360 | KEN |
| Yeast, baking powder, bicarbonate | 0 | KEN | 0 | KEN | 0 | KEN | 0 | WAF | 0 | WAF | 0 | WAF | 0 | KEN | 9.7 | KEN | 0.9 | KEN | 179 | KEN |
| Tomato sauce (bottled) | 22 | KEN | 0 | KEN | 0.10 | KEN | 1.3 | WAF | 0.63 | WAF | 97 | KEN | 0 | KEN | 0.7 | KEN | 0.2 | KEN | 115 | KEN |
| Hot sauce (Nali, etc) | 22 | KEN | 0 | KEN | 0.10 | KEN | 1.3 | UK | 0.63 | UK | 97 | KEN | 0 | KEN | 0.7 | KEN | 0.2 | KEN | 115 | KEN |
| Jam, jelly | 2 | WAF | 0 | WAF | 0 | WAF | 0 | WAF | 0.03 | WAF | 2 | WAF | 0 | WAF | 1.0 | WAF | 0.2 | WAF | 281 | WAF |
| Sweets, candy, chocolate | 21 | WAF | 0.05 | WAF | 0.08 | WAF | 0.7 | WAF | 0.04 | WAF | 12 | WAF | 0 | WAF | 4.3 | WAF | 1.4 | WAF | 532 | WAF |
| Honey | 0 | KEN | 0 | KEN | 0 | KEN | 0 | WAF | 0.02 | WAF | 2 | KEN | 0 | KEN | 0.6 | KEN | 3.0 | KEN | 522 | KEN |
| Maize- boiled/roasted (vendor) | 0 | MWI | 0.08 | MWI | 0.03 | MWI | 0.6 | MWI | 0.05 | MWI | 7 | MWI | 0 | MWI | 1.1 | MWI | 0.8 | MWI | 133 | MWI |
| Chips (vendor) | 0 | KEN | 0.19 | MWI | 0.01 | MWI | 2.0 | MWI | 0.30 | MWI | 3 | MWI | 0 | MWI | 1.0 | MWI | 0.4 | MWI | 264 | MWI |
| Cassava (vendor) | 1 | MWI | 0.03 | MWI | 0.04 | MWI | 0.4 | MWI | 0.06 | MWI | 14 | MWI | 0 | MWI | 0.6 | MWI | 0.6 | MWI | 146 | MWI |
| Eggs – boiled (vendor) | 74 | MWI | 0.12 | MWI | 0.42 | MWI | 0.1 | MWI | 0.04 | MWI | 38 | MWI | 1.8 | MWI | 2.0 | MWI | 1.3 | MWI | 164 | MWI |
| Chicken (vendor) | 13 | MWI | 0.06 | MWI | 0.05 | MWI | 2.2 | MWI | 0.14 | MWI | 3 | MWI | 0.1 | MWI | 2.9 | MWI | 2.0 | MWI | 157 | MWI |
| Meat (vendor) | 55 | MWI | 0.12 | MWI | 0.22 | MWI | 6.9 | MWI | 0.37 | MWI | 4 | MWI | 1.6 | MWI | 5.2 | MWI | 3.1 | MWI | 351 | MWI |
| Fish (vendor) | 54 | MWI | 0.08 | MWI | 0.11 | MWI | 1.1 | MWI | 0.13 | MWI | 11 | MWI | 0.1 | MWI | 4.0 | MWI | 0 | MWI | 146 | MWI |
| Mandazi (vendor) | 24 | MWI | 0.12 | MWI | 0.14 | MWI | 0.4 | MWI | 0.05 | MWI | 11 | MWI | 0.3 | MWI | 2.0 | MWI | 0.3 | MWI | 211 | MWI |
| Samosa (vendor) | 138 | KEN | 0.46 | KEN | 0.31 | WAF | 3.2 | UK | 0.15 | UK | 44 | UK | 0.3 | KEN | 3.8 | KEN | 1.8 | UK | 325 | KEN |
| Meal eaten at restaurant | 13 | MWI | 0.06 | MWI | 0.05 | MWI | 2.2 | MWI | 0.14 | MWI | 3 | MWI | 0.1 | MWI | 2.9 | MWI | 2.0 | MWI | 157 | MWI |
| Boiled sweet potato (vendor) | 7 | MWI | 0.07 | MWI | 0.01 | MWI | 0.7 | MWI | 0.09 | MWI | 8 | MWI | 0 | MWI | 0.4 | MWI | 0.2 | MWI | 97 | MWI |
| Roasted sweet potato (vendor) | 2 | MWI | 0.06 | MWI | 0.03 | MWI | 0.4 | MWI | 0.18 | MWI | 27 | MWI | 0 | MWI | 1.0 | MWI | 0.8 | MWI | 89 | MWI |
| Boiled groundnut (vendor) | 0 | MWI | 0.47 | MWI | 0.08 | MWI | 8.4 | MWI | 0.32 | MWI | 60 | MWI | 0 | MWI | 2.1 | MWI | 1.4 | MWI | 324 | MWI |
| Roasted groundnuts (vendor) | 0 | MWI | 0.78 | MWI | 0.13 | MWI | 14.7 | MWI | 0.53 | MWI | 77 | MWI | 0 | MWI | 2.1 | MWI | 2.3 | MWI | 597 | MWI |
| Zikondamoyo/Nkate | 4 | MWI | 0.18 | MWI | 0.07 | MWI | 1.2 | MWI | 0.33 | MWI | 19 | MWI | 0 | MWI | 1.4 | MWI | 0.7 | MWI | 218 | MWI |
| Cassava – roasted (vendor) | 1 | MWI | 0.03 | MWI | 0.04 | MWI | 0.4 | MWI | 0.06 | MWI | 14 | MWI | 0 | MWI | 0.6 | MWI | 0.6 | MWI | 146 | MWI |
| Tea | 0 | KEN | 0 | KEN | 0.99 | KEN | 10.8 | WAF | 0 | WAF | 5 | KEN | 0 | KEN | 2.3 | KEN | 1.7 | KEN | 299 | KEN |
| Coffee | 0 | KEN | 0 | KEN | 1.00 | KEN | 42.9 | WAF | 0.03 | WAF | 7 | KEN | 0 | KEN | 3.9 | KEN | 0.7 | KEN | 311 | KEN |
| Cocoa, millo | 61 | KEN | 0.03 | KEN | 0.15 | KEN | 0.5 | UK | 0.11 | MOZ | 6 | KEN | 0 | KEN | 2.0 | KEN | 1.6 | KEN | 541 | KEN |
| Squash (Sobo drink concentrate) | 0 | WAF | 0.02 | WAF | 0.02 | WAF | 0.1 | WAF | 0.03 | WAF | 0 | WAF | 0 | WAF | 0.3 | WAF | 0 | WAF | 48 | WAF |
| Fruit juice | 1 | MWI | 0.04 | MWI | 0.02 | MWI | 0.2 | MWI | 0.01 | MWI | 21 | MWI | 0 | MWI | 0.3 | MWI | 0.1 | MWI | 83 | MWI |
| Freezes (flavored ice) | 0 | WAF | 0.02 | WAF | 0.02 | WAF | 0.1 | WAF | 0.03 | WAF | 0 | WAF | 0 | WAF | 0.3 | WAF | 0 | WAF | 48 | WAF |
| Soft drinks | 0 | WAF | 0 | WAF | 0 | WAF | 0 | WAF | 0 | WAF | 0 | WAF | 0 | WAF | 0.1 | WAF | 0.1 | WAF | 40 | WAF |
| Chibuku (traditional beer) | 0 | WAF | 0.01 | LSO | 0.03 | LSO | 0.1 | WAF | 0.05 | WAF | 6 | LSO | 0 | WAF | 0 | LSO | 0 | WAF | 16 | WAF |
| Bottled water | 0 | MWI | 0 | MWI | 0 | MWI | 0 | MWI | 0 | MWI | 0 | MWI | 0 | MWI | 0 | MWI | 0 | MWI | 0 | MWI |
| Maheu | 0 | WAF | 0.01 | WAF | 0.03 | WAF | 0.3 | WAF | 0.05 | WAF | 6 | WAF | 0 | WAF | 0.2 | WAF | 0.1 | WAF | 33 | WAF |
| Bottled/canned beer | 0 | WAF | 0.01 | WAF | 0.02 | WAF | 0.5 | WAF | 0.03 | WAF | 6 | WAF | 0 | WAF | 0 | WAF | 0 | WAF | 41 | WAF |
| Thobwa | 0 | WAF | 0.01 | WAF | 0.03 | WAF | 0.3 | WAF | 0.05 | WAF | 6 | WAF | 0 | WAF | 0.2 | WAF | 0.1 | WAF | 33 | WAF |
| Masese (traditional beer) | 0 | WAF | 0.01 | WAF | 0.03 | WAF | 0.3 | WAF | 0.05 | WAF | 6 | WAF | 0 | WAF | 0.2 | WAF | 0.1 | WAF | 33 | WAF |
| Wine or commercial liquor | 0 | KEN | 0 | KEN | 0 | KEN | 0.1 | WAF | 0.05 | MWI | 1 | KEN | 0 | KEN | 0.5 | KEN | 0.1 | KEN | 76 | KEN |
| Kachasu (locally brewed liquor) | 0 | MWI | 0.01 | UK | 0 | UK | 0 | UK | 0 | MWI | 0 | MWI | 0 | UK | 0 | UK | 0 | UK | 231 | MWI |

***Table S2.*** *Fortifiable food equivalent factors for wheat flour in wheat flour products*

| **Food item/product** | **Fortifiable food equivalent factor** | **Recipe** | **Reference** |
| --- | --- | --- | --- |
| Wheat flour | 1 | - | - |
| Bread | 0.75 | - | ^7^ |
| Buns/scones | 0.33 | 46g Milk, cow, whole, fresh  29g Wheat flour, white, raw  12g Chicken eggs, whole, raw  12g Sugar, white | ^1^ |
| Mandazi (beignets) | 0.41 | 42g Wheat flour, white, raw  27g Chicken eggs, whole, raw  20g Milk, cow, whole, fresh  11g Oats, raw  7g Cooking oil | ^1^ |

***Table S3.*** *Base parameters defining daily dietary micronutrient and energy requirements*

| **Parameters** | | **Data & assumptions** | **Reference(s)** |
| --- | --- | --- | --- |
| *Household micronutrient supply data* | | | |
| Food consumption | | Data | ^8^ |
| Micronutrient composition of food | | See Table S1 |  |
| *Adult female equivalent factors* | | | |
| Energy requirement for reference adult female (kcal) | | 2100 | ^9^ |
| Sex of household members | | Data | ^8^ |
| Age of household members | | Data | ^8^ |
| Adult male body weight (kg) | | 65 | Assumption |
| Adult female body weight (kg) | | 55.9 | ^10^ |
| Energy expenditure factor from physical activity  (per basal metabolic rate) | | 1.6 | ^9^ |
| Additional energy requirement for pregnancy (kcal) | | 300 | ^11^ |
| Additional energy requirement for lactation (kcal) | | 500 | ^12^ |
| Energy intake from breastmilk, age 3-5 months (kcal) | | 434 | ^13,14^ |
| Energy intake from breastmilk, age 6-8 months (kcal) | | 413 | ^14^ |
| Energy intake from breastmilk, age 9-11 months (kcal) | | 379 | ^14^ |
| Energy intake from breastmilk, age 12-23 months (kcal) | | 346 | ^14^ |
| *Micronutrient inadequacy thresholds (female 18-50 years)* | | | |
| Vitamin A (μg RAE) | Harmonized Average Requirement | 490 | ^15^ |
|  | Critical nutrient density (per 1000 kcal) | 239 | ^9,15^ |
| Thiamine (mg) | Harmonized Average Requirement | 0.9 | ^15^ |
|  | Critical nutrient density (per 1000 kcal) | 0.4 | ^9,15^ |
| Riboflavin (mg) | Harmonized Average Requirement | 1.3 | ^15^ |
|  | Critical nutrient density (per 1000 kcal) | 0.6 | ^9,15^ |
| Niacin (mg) | Harmonized Average Requirement | 11 | ^15^ |
|  | Critical nutrient density (per 1000 kcal) | 5.4 | ^9,15^ |
| Vitamin B6 (mg) | Harmonized Average Requirement | 1.3 | ^15^ |
|  | Critical nutrient density (per 1000 kcal) | 0.6 | ^9,15^ |
| Folate (μg) | Harmonized Average Requirement | 250 | ^15^ |
|  | Critical nutrient density (per 1000 kcal) | 122 | ^9,15^ |
| Vitamin B12 (μg) | Harmonized Average Requirement | 2 | ^15^ |
|  | Critical nutrient density (per 1000 kcal) | 1 | ^9,15^ |
| Iron (mg) | Harmonized Average Requirement | - | ^16^ |
|  | Critical nutrient density (per 1000 kcal) | - | ^9,16^ |
| Zinc (mg) | Harmonized Average Requirement | 10.2 | ^15^ |
|  | Critical nutrient density (per 1000 kcal) | 5 | ^9,15^ |

***Table S4.*** *Standard and non-standard food consumption units* *recorded in Malawi’s Fourth Integrated Household Survey*

| **Unit** | **Sizes** |
| --- | --- |
| *Standard Units* |  |
| Gram | - |
| Kilogram | - |
| Liter | - |
| Milliliter | - |
| Tablespoon | - |
| Teaspoon | - |
|  |  |
| *Non-Standard Units* |  |
| 5L Bucket (Chigoba) | - |
| 50kg bag | - |
| Basin | Small, Large |
| Bunch | Small, Medium, Large |
| Cluster | Small, Medium, Large |
| Heap | Small, Medium, Large |
| Loaf | 300g, 600g, 700g |
| No. 10 plate | Heap, flat |
| No. 12 plate | Heap, flat |
| Packet | 150g, 400g, 500g, 1kg |
| Pail | Small, Medium, Large |
| Piece | Small, Medium, Large |
| Other (specify) | - |
| Sachet/tube | 25g, 50g, 100g |
| Tin | 100g, 250g, 500g, 1kg |
| Tina bowl | Heap, flat |

# Seasonality plots for additional micronutrients

Figure S1: Thiamine

Figure S2: Riboflavin

Figure S3: Niacin

Figure S4: Vitamin B6

Figure S5: Folate

Figure S6: Vitamin B12

Figure S7: Iron

***
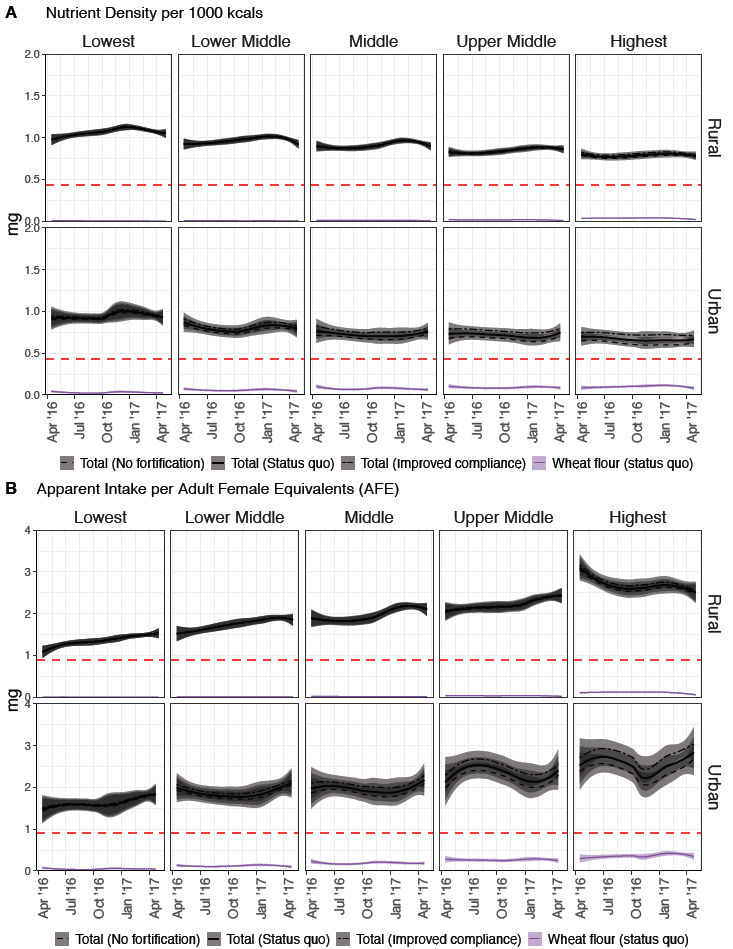
***

***Figure S1.*** *Seasonality in the (A) nutrient density and (B) apparent intake of thiamine under the three fortification scenarios in relation to the inadequacy threshold (red dotted line) by socioeconomic position (lowest to highest) between urban and rural residences.*

***
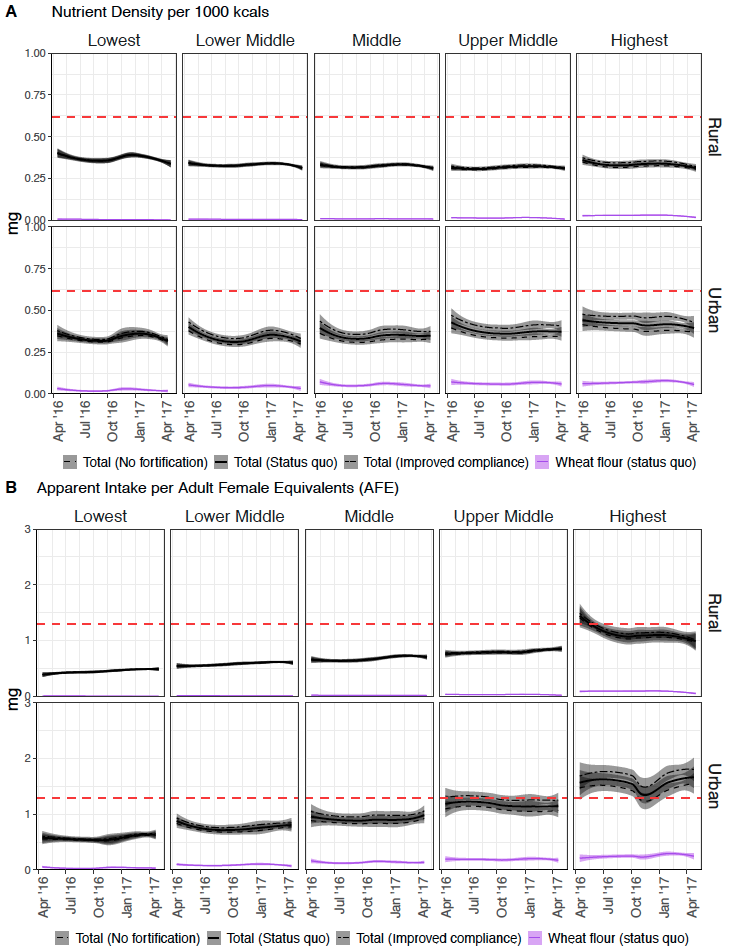
***

***Figure S2.*** *Seasonality in the (A) nutrient density and (B) apparent intake of riboflavin under the three fortification scenarios in relation to the inadequacy threshold (red dotted line) by socioeconomic position (lowest to highest) between urban and rural residences.*

***
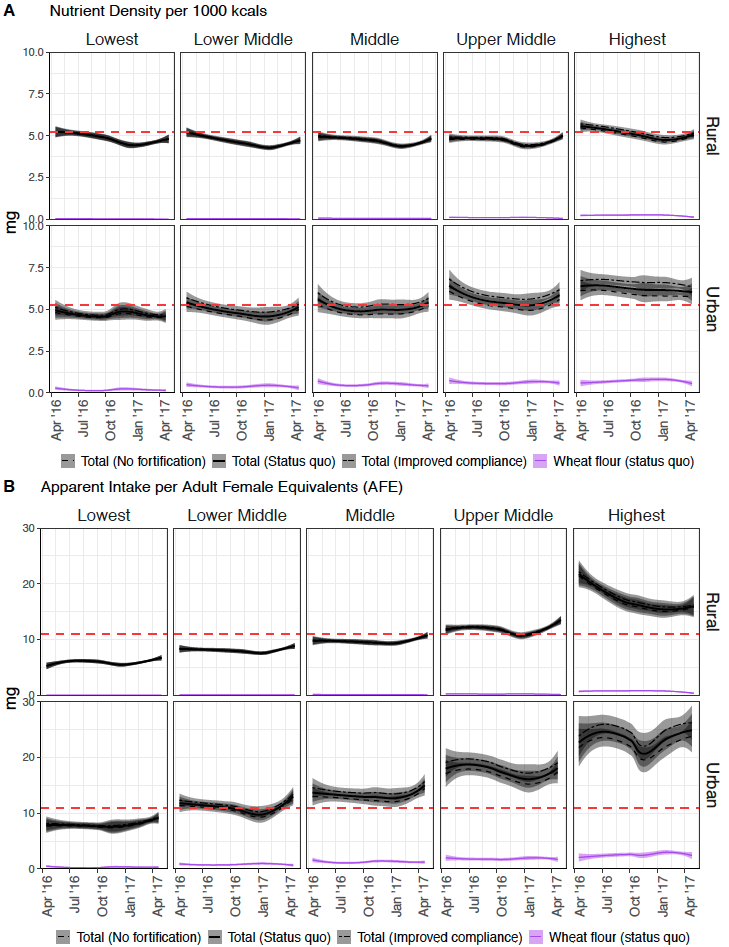
***

***Figure S3.*** *Seasonality in the (A) nutrient density and (B) apparent intake of niacin under the three fortification scenarios in relation to the inadequacy threshold (red dotted line) by socioeconomic position (lowest to highest) between urban and rural residences.*

***
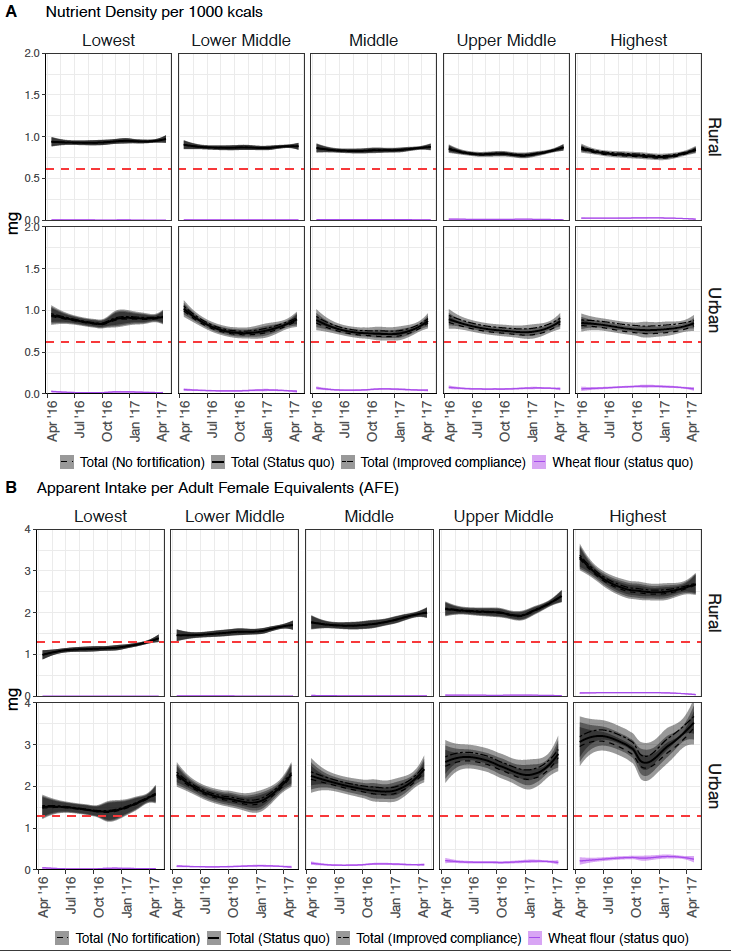
***

***Figure S4.*** *Seasonality in the (A) nutrient density and (B) apparent intake of vitamin B6 under the three fortification scenarios in relation to the inadequacy threshold (red dotted line) by socioeconomic position (lowest to highest) between urban and rural residences.*

***
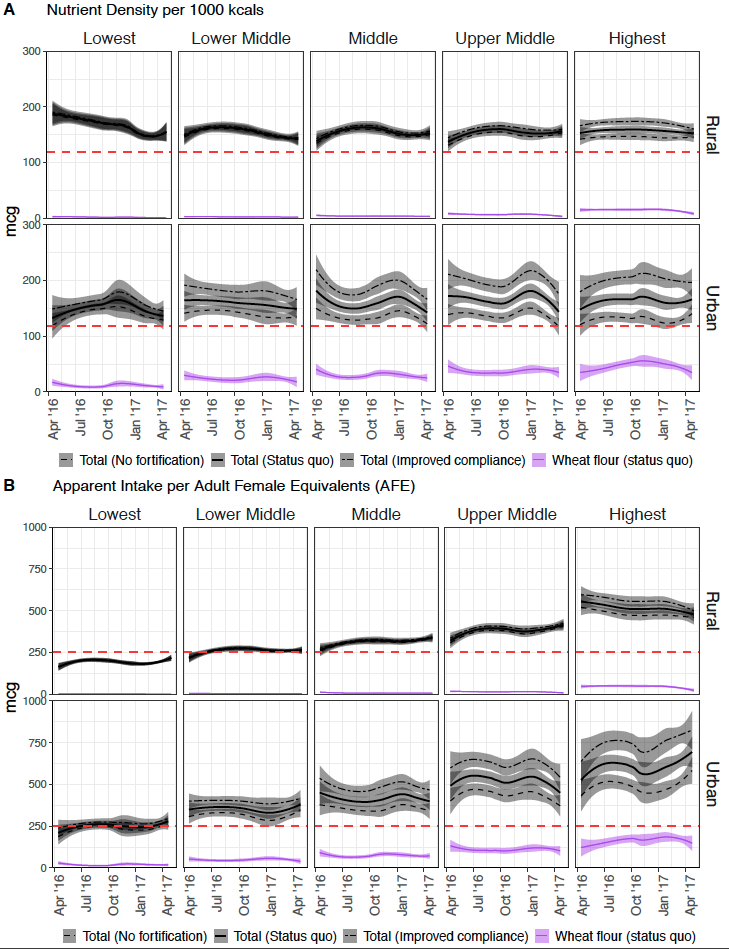
***

***Figure S5.*** *Seasonality in the (A) nutrient density and (B) apparent intake of folate under the three fortification scenarios by socioeconomic position (lowest to highest) between urban and rural residences.*

***
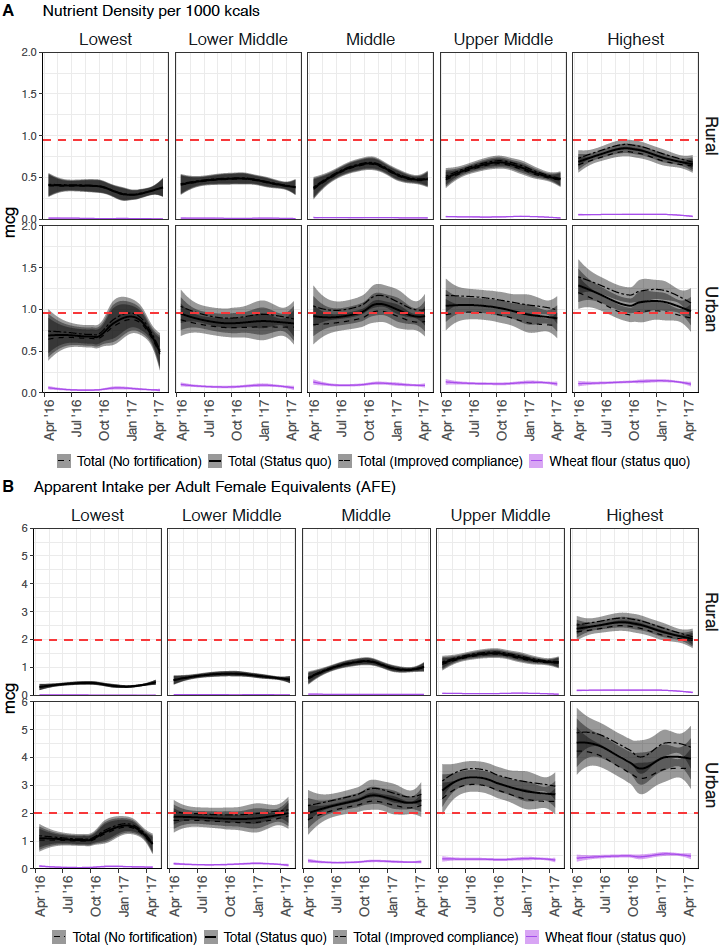
***

***Figure S6.*** *Seasonality in the (A) nutrient density and (B) apparent intake of vitamin B12 under the three fortification scenarios in relation to the inadequacy threshold (red dotted line) by socioeconomic position (lowest to highest) between urban and rural residences.*

***
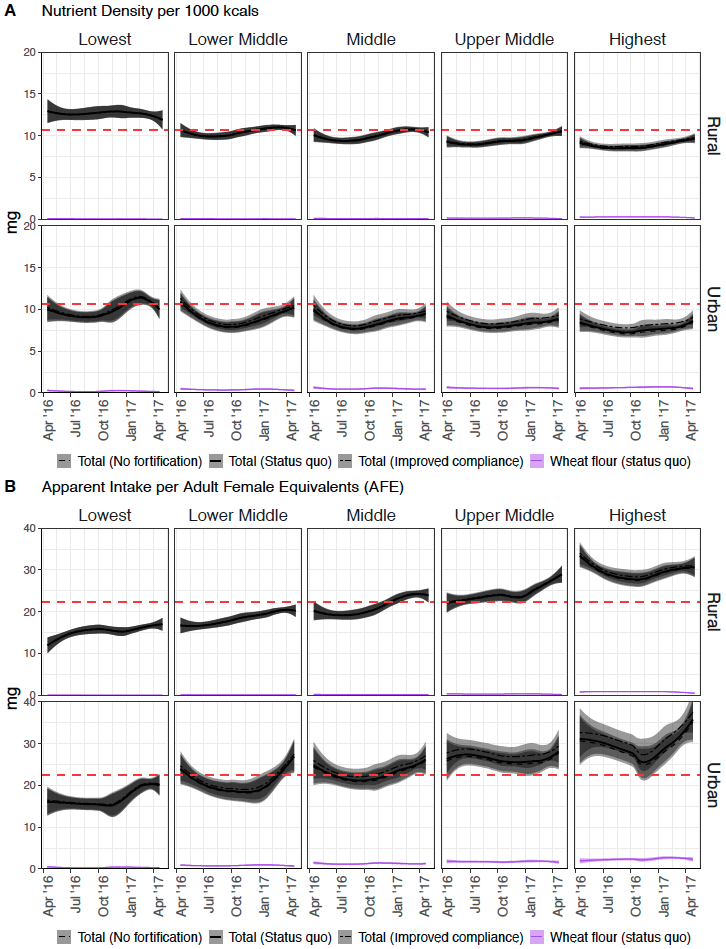
***

***Figure S7.*** *Seasonality in the (A) nutrient density and (B) apparent intake of iron under the three fortification scenarios in relation to the inadequacy threshold (red dotted line) by socioeconomic position (lowest to highest) between urban and rural residences. While the threshold for inadequacy is present, estimating dietary iron inadequacy requires the full probabilistic approach since iron requirements for menstruating women are non-parametric.*

# Prevalence of apparent vitamin A intake above the Harmonized-Upper Limit

**Table S5.** Prevalence of households exceeding the daily Harmonized-Upper Limit for vitamin A apparent intake across large-scale food fortification scenarios by sub-population.

| Population | Households, *n* | Excessive apparent intake prevalence, % | | |
| --- | --- | --- | --- | --- |
|  |  | *No fortification* | *Status quo* | *Improved compliance* |
| **National (total)** | **12,447** | **1** | **1** | **3** |
| *Geography by administrative region* | | | | |
| North | 2491 | 0 | 1 | 3 |
| Center | 4220 | 1 | 1 | 3 |
| South | 5736 | 1 | 1 | 3 |
| *Residence & socioeconomic position (SEP) by quintile of total annual household expenditure per capita* | | | | |
| **Rural (total)** | **10,175** | **1** | **1** | **2** |
| Lowest SEP | 2035 | 0 | 0 | 0 |
| Lower Middle SEP | 2035 | 0 | 0 | 0 |
| Middle SEP | 2035 | 0 | 0 | 0 |
| Upper Middle SEP | 2035 | 1 | 1 | 1 |
| Highest SEP | 2035 | 1 | 3 | 8 |
| **Urban (total)** | **2272** | **0** | **2** | **6** |
| Lowest SEP | 455 | 0 | 0 | 0 |
| Lower Middle SEP | 454 | 1 | 1 | 2 |
| Middle SEP | 455 | 0 | 1 | 2 |
| Upper Middle SEP | 454 | 0 | 2 | 6 |
| Highest SEP | 454 | 1 | 5 | 19 |


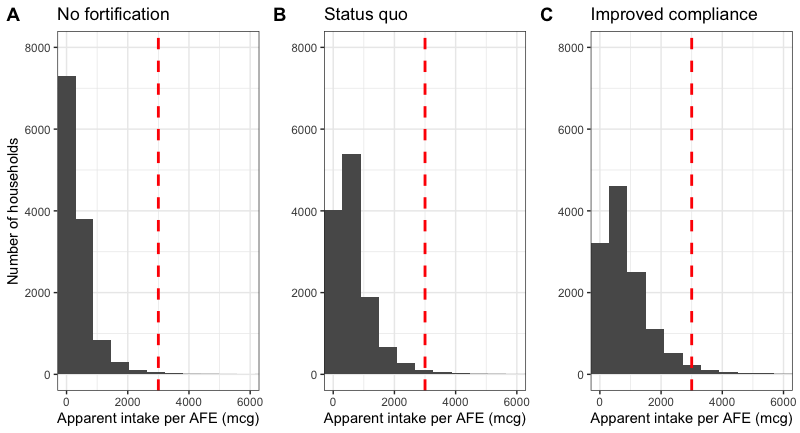


**Figure S8.** Histogram of apparent vitamin A intake per adult female equivalent in relation to the Harmonized-Upper Limit for daily vitamin A intake (dotted red line) under the three large-scale food fortification scenarios.

# References

1. Van Graan A., J. Chetty, M. Jumat, *et al.* 2019. “*Malawian Food Composition Table*.” Lilongwe.

2. Food and Agriculture Organization of the United Nations & Government of Kenya. 2018. “*Kenya Food Composition Tables*.” Nairobi.

3. Lephole M.M., M.C. Khaketla & M.E. Monoto. 2006. “*Composition of Lesotho Foods*.” Maseru.

4. Korkalo L., H. Hauta-alus & M. Mutanen. 2011. “*Food Composition Tables for Mozambique*.” Maputo.

5. Vincent A., F. Grande, E. Compaoré, *et al.* 2020. “*FAO/INFOODS Food Composition Table for Western Africa*.” Rome.

6. McCance R. & E. Widdowson. 2020. “*McCance and Widdowson’s The Composition of Foods Integrated Dataset*.” London.

7. Engle-Stone R., M. Nankap, A.O. Ndjebayi, *et al.* 2014. Simulations based on representative 24-h recall data predict region-specific differences in adequacy of vitamin a intake among Cameroonian women and young children following large-scale fortification of vegetable oil and other potential food vehicles. *J. Nutr.* **144**: 1826–34.

8. National Statistical Office & The World Bank. 2017. “*Fourth Integrated Household Survey of Malawi*.” Lilongwe.

9. FAO/WHO/UNU. 2004. Human energy requirements. Report of a Joint FAO/WHO/UNU Expert Consultation: Rome, 17–24 October 2001. *AO food Nutr. Tech. Rep. Ser.*

10. National Statistics Office (NSO). 2017. “*Malawi Demographic and Health Survey 2015-16*.” Zomba.

11. Kominiarek M.A. & P. Rajan. 2016. Nutrition Recommendations in Pregnancy and Lactation. *Med. Clin. North Am.* **100**: 1199–1215.

12. US Centers for Disease Control and Prevention. Accessed October 8, 2020. https://www.cdc.gov/breastfeeding/breastfeeding-special-circumstances/diet-and-micronutrients/maternal-diet.html.

13. Walters C.N., H. Rakotomanana, J.J. Komakech, *et al.* 2019. Maternal determinants of optimal breastfeeding and complementary feeding and their association with child undernutrition in Malawi (2015-2016). *BMC Public Health* **19**: 1503.

14. WHO Programme of Nutrition. 1998. “*Complementary feeding of young children in developing countries: a review of current scientific knowledge*.” Geneva.

15. Allen L.H., A.L. Carriquiry & S.P. Murphy. 2020. Proposed Harmonized Nutrient Reference Values for Populations. *Adv. Nutr.* **11**: 469–483.

16. Allen, L., de Benoist, B., Dary, O. & Hurrell R. 2006. “*Guidelines on food fortification with micronutrients*.” Geneva.

17. Weisell R. & M.C. Dop. 2012. The adult male equivalent concept and its application to Household Consumption and Expenditures Surveys (HCES). *Food Nutr. Bull.* **33**: S157-62.
